# Repair of DNA and protein damages caused by formaldehyde improves methanol assimilation

**DOI:** 10.1016/j.fmre.2025.05.007

**Published:** 2025-05-17

**Authors:** Cheng Zhu, Yun Chen, Wenjie Sun, Jian Li, Haiyan Liu, Jiahui Peng, Yanfen Bai, Ramon Gonzalez, Zaigao Tan

**Affiliations:** a State Key Laboratory of Microbial Metabolism, Shanghai Jiao Tong University, Shanghai 200240, China; b Department of Bioengineering, School of Life Sciences and Biotechnology, Shanghai Jiao Tong University, Shanghai 200240, China; c Shenzhen Institute of Advanced Technology, Chinese Academy of Sciences, Shenzhen 518000, China; d Department of Chemical, Biological and Materials Engineering, University of South Florida, Tampa, FL 33620, USA

**Keywords:** Formaldehyde tolerance, Methanol utilization, DNA-protein crosslinks, Protein damage, Biosynthesis

## Abstract

Methanol is regarded as a next-generation feedstock. However, the efficiency of synthetic methylotrophs remains suboptimal compared to their preferred carbon sources. In this study, we conducted a comprehensive investigation into the microbial assimilation of methanol, revealing that this process is impeded by formaldehyde toxicity. By utilizing DNA-protein cross-links (DPC) protease GCNA1 from *Caenorhabditis elegans* and thioproline aminopeptidase PepP from *Escherichia coli*, we effectively mitigated the formaldehyde-causing DNA and protein damages. Integration of these damage-repair enzymes in methanol-assimilating *E. coli* strains led to a substantial improvement in methanol consumption. Specifically, the engineered *E. coli* strain demonstrated a methanol consumption amount of up to 440 mM (∼14.1 g/L), with an average consumption rate of 0.229 mM/h. This represents a remarkable 50-fold increase compared to the control strain. Notably, this achievement stands out as the highest methanol consumption level observed among all methanol-assimilating *E. coli* strains, highlighting the pivotal role of alleviating formaldehyde cytotoxicity in enhancing methanol assimilation efficiency.

## Graphical abstract

**Figure:**
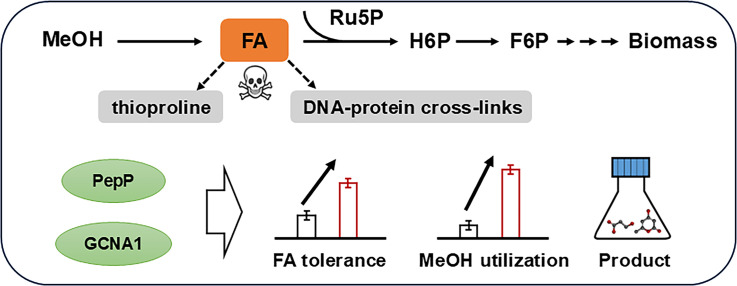

## 1 Introduction

**Fig. 1:**
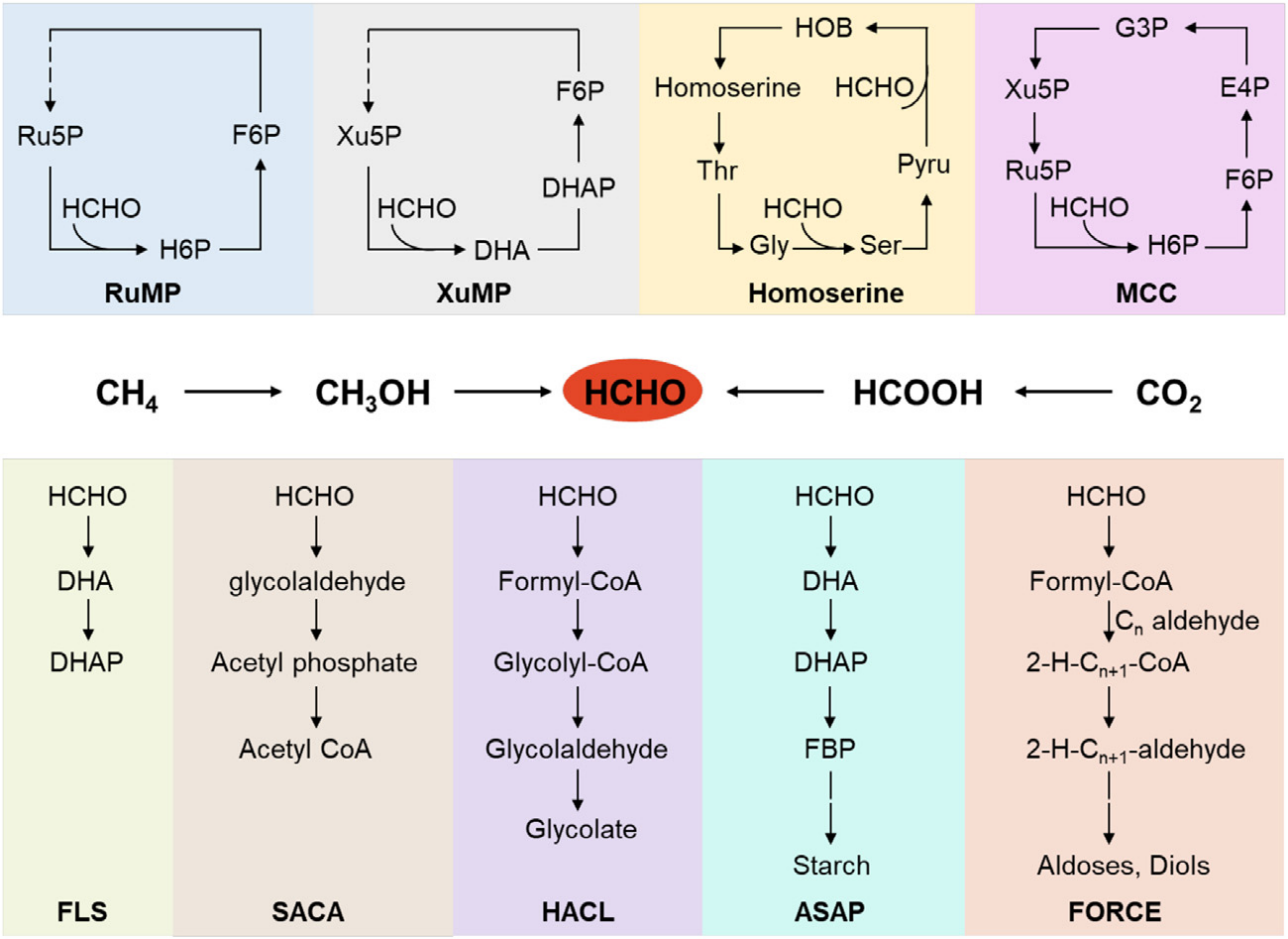
**Formaldehyde (HCHO) as an important intermediate during methylotrophic metabolisms.** Formaldehyde functions as a central intermediary in a variety of natural, modified, or synthetic methylotrophic pathways. RuMP, ribulose monophosphate; H6P, hexulose 6-phosphate; F6P, fructose 6-phosphate; Ru5P, ribulose 5-Phosphate; XuMP, xylulose monophosphate; DHA, dihydroxyacetone; DHAP, dihydroxyacetone phosphate; F6P, fructose 6-phosphate; Xu5P, xylulose 5-phosphate; Pyru, pyruvate; HOB, 4‑hydroxy-2-oxobutanoate; MCC, methanol condensation cycle; E4P, Erythrose-4-phosphate; G3P, Glyceraldehyde 3 phosphate; FLS, formolase pathway; SACA, synthetic acetyl-CoA pathway; HACL, 2-hydroxyacyl-CoA-lyase pathway; ASAP, artificial starch anabolic pathway; FBP, fructose-1;6-bisphosphate; FORCE; formyl-CoA elongation pathway.

Climate change and the unsustainability of fossil fuels necessitate the transition to cleaner energy sources, including methanol as a viable fuel option [1]. Methanol, one of the simplest molecules for energy storage [1], is an ideal and renewable feedstock for chemical manufacturing due to its abundance and compatibility with existing transportation and biomanufacturing infrastructure as a liquid [2]. The production of substantial quantities of methanol from CO_2_ through photocatalytic or electrochemical reduction processes offers a promising pathway for establishing process chains that yield value-added chemicals with a minimal or nearly zero CO_2_ footprint, aligning with carbon neutrality goals (Fig. 1) [2].

Mitigation of greenhouse gas (GHG) emissions has sparked significant interest in utilizing methanol as a carbon source for bioprocessing applications [2]. Native methylotrophic microbes such as *Bacillus methanolicus* and *Methylorubrum extorquens* can naturally assimilate methanol, making them attractive biocatalysts for this purpose [3,4]. However, significant challenges remain in the utilization of native methylotrophs, including an unclear genetic framework, a lack of reliable genetic manipulation tools, and a limited spectrum of products [5]. In comparison, researchers have been focusing on engineering synthetic methylotrophic microbes by introducing methylotrophic pathways into model microbes, such as *Escherichia coli*. Nevertheless, these synthetic methylotrophs are currently unsuitable for industrial applications due to their inefficient methanol conversion, which has been associated with low activity of methylotrophic pathways. To this end, extensive efforts have been directed to rationally engineer natural and/or design artificial methylotrophic pathways [[6], [7], [8], [9], [10], [11], [12], [13], [14], [15], [16]]. However, the efficiency of synthetic methylotrophs still falls short when compared to their preferred carbon sources, such as sugars. For instance, in methanol-metabolizing *E. coli* strains, all rational engineering works (without adaptive laboratory evolution) have led to methanol assimilation levels within the 10–150 mM range, with rates varying from 0.1 to 0.8 mM/h (Table S1).

Formaldehyde serves as a central intermediate in most methylotrophic pathways [17,18], e.g., RuMP pathway [19], XuMP pathway [20], homoserine cycle [21], MCC pathway [10], FLS pathway [15], SACA pathway [13], HACL pathway [16], ASAP pathway [11], and FORCE pathway [12,16] (Fig. 1). Among these, the RuMP pathway demonstrates a notable advantage in both energy utilization and carbon fixation efficiency. It can effectively convert methanol to formaldehyde and further transform it into central carbon metabolic intermediates [19,22]. Nevertheless, formaldehyde easily generates chromosomal DNA damages, including DNA-protein cross-links (DPC), DNA strand cross-links, and DNA strand breaks [23]. Besides, formaldehyde also generates severe protein damage by inducing protein crosslinking and forming toxic compounds by reacting spontaneously with the specific groups of proteins. The cytotoxicity of formaldehyde has not been efficiently mitigated, thereby impeding efficient methanol assimilation and restricting biomass accumulation to relatively low levels [24,25]. To this end, we aim to alleviate formaldehyde toxicity by repairing formaldehyde-generating damage. This repair process resulted in a notable enhancement in methanol assimilation, as well as the production of methanol-based products.

## 2 Materials and methods

### 2.1 Strains and plasmids

All plasmids and strains utilized in this study are comprehensively described in Table S2-S4. *E. coli* MG1655 served as the initial strain for the tolerance test, and MG1655(DE3) was employed as the starting strain for methanol assimilation and product synthesis. The CRISPR-Cas9 method [26] was applied for chromosomal editing, encompassing the incorporation of diverse heterologous genes and the knockout genes of *frmA, rpiA*, and *rpiB*. The one-step inactivation technique (FLP-FRT) [27] was utilized for knocking out the *cyaA* gene. In this study, except for genes originating from *E. coli* and *Saccharomyces cerevisiae*, the remaining foreign genes underwent codon optimization before being inserted into the *mgsA* site of *E. coli*. All the protein information utilized to alleviate DPC and protein damage is provided in Table S5-S6. All gene expression is regulated by the artificial part M1–93 [28]. For methanol assimilation, the *mdh* gene from *C. necator* and the *hps* and *phi* genes from *B. methanolicus* were inserted into the pCDF-duet plasmid to construct pCDF-RuMP. For 3-HP fermentation, the *mcrC* and *mcrN* genes from *Chloroflexus aurantiacus* were incorporated into the pET-duet plasmid to construct pET-*mcrC-mcrN*. For TAL fermentation, the *bktB* gene from *C. necator* was integrated into the pTrc99a plasmid, resulting in pTrc99a-*bktB*.

Strain MPD4 was generated through the incorporation of the *pepP* and *gcna1* genes. The process began with the MG1655(DE3) Δ*frmA* Δ*rpiA* strain, in which the *pepP* and *gcna1* genes were integrated at the *mgsA* site using a ribosome binding site (RBS) linker sequence "GTTTAAACCAGGAGAATTAAA". Following this, the *rpiB* and *cyaA* genes were sequentially knocked out, and plasmid pCDF-RumP was introduced to produce strain MPD4.

### 2.2 Assessment of solvent tolerance of engineered strains

Solvent tolerance tests were conducted in 200 μL MOPS minimal medium (comprising 40 mM MOPS, 4 mM tricine, 0.01 mM FeSO_4_, 9.5 mM NH_4_Cl, 0.276 mM K_2_SO_4_, 0.5 μM CaCl_2_, 0.525 mM MgCl_2_, 50 mM NaCl, 0.292 nM (NH_4_)_2_MoO_4_, 40 nM H_3_BO_3_, 3.02 nM CoCl_2_, 0.962 nM CuSO_4_, 8.08 nM MnCl_2_, 0.974 nM ZnSO_4_, and 1.32 mM K_2_HPO_4_) [29] supplemented with 2% (wt/v) glucose in a clear-bottom 96-well plate. The experiments were carried out at 37 °C with an initial pH of 7.0. The specific growth rate μ (*h*^−1^) was calculated by fitting the equation OD_550,_*_t_* = OD_550,0_ e^μt^ to the exponential growth phase. All estimated μ values demonstrated an *R*^2^ value of at least 0.95. The increased specific growth rate was calculated by subtracting the difference between the μ of the engineered strain and the μ of the control strain, then dividing by the μ of the control strain.

Moreover, to ascertain the threshold of the strain's formaldehyde tolerance, the selected strains were cultivated in 5 mL of MOPS minimal medium in 50 mL centrifuge tubes, supplemented with 2% (wt/v) glucose and 1.2 mM formaldehyde. The cultures were maintained at 37 °C under constant agitation at 220 rpm throughout the experiment.

### 2.3 Determination of protein concentration

Protein concentration in the supernatant following formaldehyde treatment was quantified using the Bradford assay [30]. *E. coli* MG1655 was cultured in 10 mL MOPS medium supplemented with 2% glucose at 37 °C and 220 rpm until OD_550_ reached between 1.5 and 2.0. Subsequently, formaldehyde was introduced to the centrifuge tube at varying final concentrations. The culture was maintained under the same conditions for an additional 10 h, after which cells were harvested by centrifugation at 12,000 rpm and washed twice with PBS buffer (pH 7.5). The resultant pellet was then resuspended in 2 mL of PBS buffer and subjected to sonication for 5 min (power: 150 W, 2 s on, 3 s off). The supernatant was collected by centrifugation at 12,000 rpm for 5 min. To determine the protein concentration, G250 solution was added to the supernatant along with standard protein samples, and the absorbance at 595 nm was measured. The target protein concentration was subsequently calculated based on these measurements.

### 2.4 Methanol assimilation fermentation experiment

The strains were cultured overnight in LB medium containing 50 μg/mL kanamycin and 50 μg/mL spectinomycin. Following this incubation, the bacteria cells were harvested by centrifugation, washed twice with MOPS minimal medium, and subsequently inoculated into MOPS minimal medium supplemented with 2% (wt/v) casamino acids (Sangon Biotech), along with 50 μg/mL kanamycin, 50 μg/mL spectinomycin, 0.1 mM isopropyl β-d-1-thiogalactopyranoside (IPTG), and the desired concentration of methanol and xylose. The fermentation was initiated with an initial OD_550_ of 0.1. The experiment was conducted at 37 °C and a shaking speed of 220 rpm.

For the methanol and xylose consumption assay and fermentation in baffled flasks, the MOPS minimal medium was supplemented with 600 mM methanol, 50 mM xylose, 2% (wt/v) casamino acids, 50 μg/mL kanamycin, 50 μg/mL spectinomycin, and 0.1 mM IPTG. In the fermentation experiment involving only the feeding of xylose, 30 mM xylose was added daily starting from day 2. In the fermentation experiment with simultaneous feeding of methanol and xylose, 30 mM xylose was added daily from day 2, followed by the addition of 100 mM methanol daily commencing on day 5. Samples were collected for OD_550_ measurement and subsequent analysis using HPLC.

### 2.5 Determination of DNA–protein crosslink

For the measurement of DNA-protein crosslinks (DPCs) using the KCl-SDS method [31,32], strains were cultured overnight and subsequently treated with varying concentrations of formaldehyde. Following treatment, 0.5 mL of the cell culture was harvested and subjected to two washes with PBS buffer (pH = 7.5). The cells were then resuspended in 0.5 mL of PBS buffer at pH 7.5. Subsequently, 0.5 mL of a 2% SDS solution was introduced to the cell suspension and gently vortexed. The mixture was then heated at 65 °C for 10 min to facilitate cell lysis. To this, 100 μL of 1 M KCl in 0.2 M Tris–HCl at pH 7.5 was added, followed by passing the solution six times through a 1-mL polypropylene pipette tip to promote shearing of DNA to a consistent length. Adding SDS is vital as it can bind to proteins but not DNA. Consequently, the DPCs and other associated proteins form a precipitate, leaving the free DNA in the supernatant. Subsequently, the SDS-*K*^+^ precipitate was allowed to form by cooling on ice for 5 min and was then collected by centrifugation at 13,000 rpm for 5 min at 4 °C.

The supernatant containing free DNA was carefully transferred to a new tube. The pellet was washed three times by resuspending it each time in 1 mL of washing buffer (comprising 0.1 M KCl, 0.1 mM EDTA, 20 mM Tris–HCl at pH 7.5), followed by heating at 65 °C for 5 min and subsequent centrifugation as previously described. The supernatant obtained from each wash step was combined with the contents of the previously reserved new tube containing free DNA. The pellet resulting from the wash steps was then resuspended in 0.5 mL of washing buffer, following which 0.5 mL of protease K (at a concentration of 0.4 mg/mL) was added, and the mixture was allowed to digest for 3 h at 50 °C. Subsequently, the mixture was cooled on ice for 5 min and centrifuged at 13,000 rpm for 10 min at 4 °C. The supernatant, which contained DNA involved in DPC formation, was carefully transferred to another fresh tube.

To enable quantification, DNA standard samples with final concentrations ranging from 0 to 5000 ng/mL were prepared. All samples were treated with freshly prepared fluorescent dye Hoechst 33258 and incubated in the dark for 30 min. The fluorescence intensity of the samples was measured using a microplate reader with excitation at 350 nm and emission at 450 nm. Quantitative determination of DNA content in the samples was achieved by comparison with the corresponding DNA standards. The DPC value was computed as the ratio of cross-linked DNA to total DNA, where the total DNA comprised the sum of cross-linked DNA and free DNA.

### 2.6 Determination of intracellular formaldehyde concentration

To quantify intracellular formaldehyde, the Nash reaction was modified based on the methodology described by Woolston et al. [33]. Cells were centrifuged at 12,000 rpm and washed twice with PBS buffer (pH = 7.5). The resultant pellet was then resuspended in 2 mL of PBS buffer and sonicated for 5 min (power: 150 w, 2 s on, 3 s off). A 150 μL aliquot of this resuspension was transferred to a new tube, followed by the addition of 150 μL of Nash reagent (composed of 5 M ammonium acetate and 50 mM acetylacetone). The mixture was incubated for 1 hour at 37 °C, after which absorbance measurements were recorded at 412 nm. A fresh standard curve was established for each assay, encompassing a concentration range from 0 to 1 mM.

### 2.7 Assessment of methanol and xylose consumption

Samples were collected for OD_550_ measurement and evaluation of methanol and xylose utilization. An Agilent HPX-87H (300 × 7.8 mm) column was employed to quantify methanol and xylose concentrations. Briefly, a mobile phase of 5 mM sulfuric acid was utilized at a flow rate of 0.6 mL/min over a 30-minute run time. The column temperature was maintained at 65 °C, and detection was performed using a refractive index detector (RID). Methanol exhibited a retention time of 18.9 min, while xylose showed a retention time of 9.3 min.

### 2.8 13C labeling experiment

For the analysis of ^13^C-labelled metabolites in the central metabolic pathway, strain MPD4 was cultivated in LB medium overnight. The cells were subsequently washed twice with MOPS medium and then transferred into MOPS medium with 50 mM xylose, 2% casein hydrolysate, 100 mM ^13^C-labelled methanol, as well as 50 μg/mL kanamycin, 50 μg/mL spectinomycin, and 0.1 mM IPTG. The initial OD_550_ was adjusted to 0.1. After 8 days, samples were collected for ^13^C metabolite tracer analysis. The labelled amino acid and central metabolites were then analyzed by liquid chromatography mass spectrometry (LC-MS) following the procedures described in a previous study [34].

### 2.9 Product fermentation

Strains containing the pET-*mcrC-mcrN* or pTrc99a-*bktB* plasmid were inoculated into LB medium supplemented with 50 μg/mL kanamycin, 50 μg/mL spectinomycin, and 50 μg/mL carbenicillin and cultured overnight. Following this, the bacterial cells were harvested by centrifugation, washed twice with MOPS minimal medium, and subsequently inoculated into MOPS minimal medium enriched with 500 mM methanol, 50 mM xylose, 2% (wt/v) casamino acids, as well as 50 μg/mL kanamycin, 50 μg/mL spectinomycin, 50 μg/mL carbenicillin, and 0.1 mM IPTG, achieving an initial OD_550_ of 0.1 for fermentation. The experiment was conducted at 37 °C with shaking at 220 rpm. Daily samples were collected to measure OD_550_, methanol, xylose, and their corresponding products. During the production of TAL, an additional 30 mM of xylose was supplemented daily upon exhaustion of xylose in the medium.

### 2.10 3-HP and TAL quantification

Samples were collected every 24 h to determine the concentration of corresponding products. The quantification of 3-HP was conducted using an Agilent HPX-87H (300 × 7.8 mm) column. A mobile phase of 5 mM sulfuric acid at a flow rate of 0.6 mL/min was employed for a 30-minute run. The column temperature was maintained at 65 °C, and detection was carried out using a diode array detector set at 210 nm. TAL was extracted by ethyl acetate, followed by derivatization using pyridine and *N,O*-Bis(trimethylsilyl) trifluoroacetamide (BSTFA). TAL was identified by GC–MS based on a previously established protocol [35].

### 2.11 Statistical analysis

The statistical significance of all data in this study was analyzed using the two-tailed *t*-test method, with a *p*-value < 0.05 considered statistically significant.

## 3 Results

### 3.1 Methanol assimilation is impeded by formaldehyde cytotoxicity

**Fig. 2:**
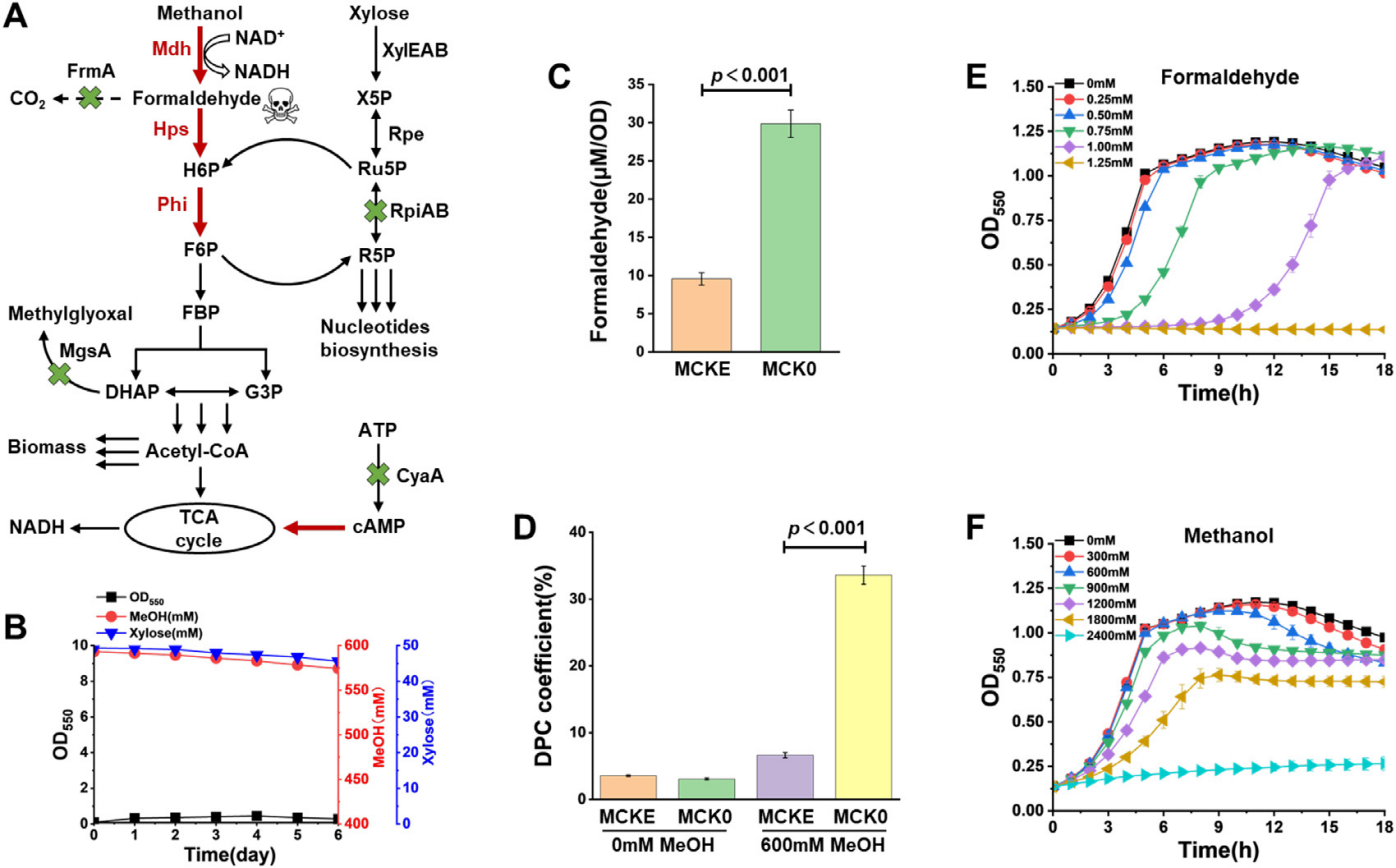
**Methanol assimilation is impeded by formaldehyde cytotoxicity.** (a) Schematic illustration of the genetic manipulations performed on the methanol-utilizing *E. coli* chassis cell. (b) Growth dynamics and metabolic utilization of methanol and xylose by strain MCK0 during cultivation in 40 mL MOPS medium containing 600 mM methanol and 50 mM xylose in 250 mL baffled flasks. (c) Measurement of intracellular formaldehyde (μM/OD_550_) in MCKE and MCK0 cultivated in LB medium with 600 mM methanol and 0.1 mM IPTG. (d) DPC coefficients in MCKE and MCK0 strains cultivated in LB medium with 0.1 mM IPTG, and 0 mM or 600 mM methanol. (e) & (f) Depicts the growth response to varying concentrations of formaldehyde (E, 0 to 1.25 mM), and methanol (F, 0 to 2400 mM) in MOPS supplemented with 2% (wt/v) glucose, conducted in a clear-bottom 96-well plate incubated at 37 °C and an initial pH of 7.0. Values are the average of at least three biological replicates, and error bars indicate standard deviation.

We first introduced the methanol assimilation pathway in the starting strain *E. coli* MG1655(DE3) Δ*mgsA* with inactivation of the toxic methylglyoxal formation pathway [36]. The ribulose monophosphate (RuMP) pathway, a key methylotrophic pathway, consisting of methanol dehydrogenase (MDH), 3-hexulose-6-phosphate synthase (HPS), and 6-phospho-3-hexuloisomerase (PHI) [34], was recruited (Fig. 2a). In this pathway, MDH first catalyzes the oxidation of methanol to formaldehyde, which is then assimilated by HPS and PHI into the central carbon metabolism of *E. coli*. To enable this conversion process, the *mdh* gene from *Cupriavidus necator* [37] and the *hps* and *phi* genes from *Bacillus methanolicus* [19] were incorporated and assembled into an empty pCDFduet-1 vector, yielding the pCDF-*mdh*-*hps*-*phi* recombinant plasmid (pCDF-RuMP).

Endogenous S-(hydroxymethyl) glutathione dehydrogenase of *E. coli* can catalyze the conversion of formaldehyde into CO_2_ [17]. To mitigate this potential loss, we deleted the *frmA* gene. Given that the RuMP pathway relies on ribulose 5-phosphate (Ru5P) as a precursor [38], we enhanced the availability of this substrate by supplementing the strain with xylose. As wild-type *E. coli* can metabolize xylose as its primary carbon source, we further knocked out the *rpiA* and *rpiB* genes to block the conversion of Ru5P to ribose 5-phosphate (R5P), thereby channeling xylose assimilation through the RuMP pathway [17]. Additionally, we disrupted the *cyaA* gene (which encodes adenylate cyclase) to potentially reduce methanol utilization by downregulating enzymes in the TCA cycle [38] (Fig. 2a).

We transformed the pCDF-RuMP recombinant plasmid into the engineered strain, yielding the MCK0 strain (MG1655(DE3) Δ*mgsA* Δ*frmA* Δ*rpiA* Δ*rpiB* Δ*cyaA* + pCDF-RuMP) (Table S2). The MCK0 strain was then cultured in MOPS minimal medium supplemented with a moderate concentration of 600 mM methanol (∼19.2 g/L), 50 mM xylose, and 2% (wt/v) casamino acids in shake flasks. All the methanol evaporation determination experiments were performed and can be seen in Fig. S1. The MCK0 strain demonstrated difficulty in proliferation, reaching its maximum cell mass (OD_550_ = 0.36) only after > 3 days of cultivation, and ultimately utilized only 9 mM methanol (0.3 g/L) and 4 mM xylose (0.6 g/L) within 6 days (Fig. 2b). This finding corresponds with the methanol assimilation levels observed in RuMP pathway-utilizing methanol-assimilating *E. coli* strains as reported in prior studies [6,19,24].

We hypothesized that the low growth and methanol assimilation inefficiencies were probably attributed to the accumulation of toxic formaldehyde resulting from the RuMP pathway. To test this assumption, we harvested cells from the MCK0 strain and measured their intracellular formaldehyde concentration. Specifically, the intracellular formaldehyde concentration in the MCK0 strain reached at least 29.9 μM/OD_550_, which was 3-fold higher (*P* < 0.001) compared to MCKE (containing pCDF-empty) (9.6 μM/OD_550_) (Fig. 2c). We also assessed the formation of DNA-protein cross-links (DPC) in the two strains. At 0 mM methanol concentration, no notable distinctions were observed in the DPC coefficient between the two strains. However, at 600 mM methanol, this DPC coefficient in the MCK0 strain rose to at least 34%, marking a fourfold increase (*P* < 0.001) compared to the MCKE strain (∼7%) (Fig. 2d).

To further assess the degree of formaldehyde toxicity to *E. coli*, we added exogenous formaldehyde to wild-type *E. coli* MG1655. Our findings revealed that formaldehyde exerted significant inhibitory effects on *E. coli*, with a concentration of merely 1.25 mM sufficient to hinder its growth in MOPS medium supplemented with 2% (wt/v) glucose (Fig. 2e). In parallel, we evaluated the toxicity of methanol on *E. coli*, observing only a slight inhibitory effect; *E. coli* MG1655 remained viable even at a concentration of 2400 mM methanol (Fig. 2f). Notably, the lethal concentration of formaldehyde (1.25 mM) is approximately 2000-fold lower than that of methanol (2400 mM). These results indicated that the accumulation of toxic formaldehyde in the RuMP pathway, rather than the methanol substrate per se, acts as the main factor limiting both cell growth and the efficiency of methanol assimilation in the MCK0 strain.

### 3.2 Improving methanol assimilation by repairing formaldehyde-causing DPC

**Fig. 3:**
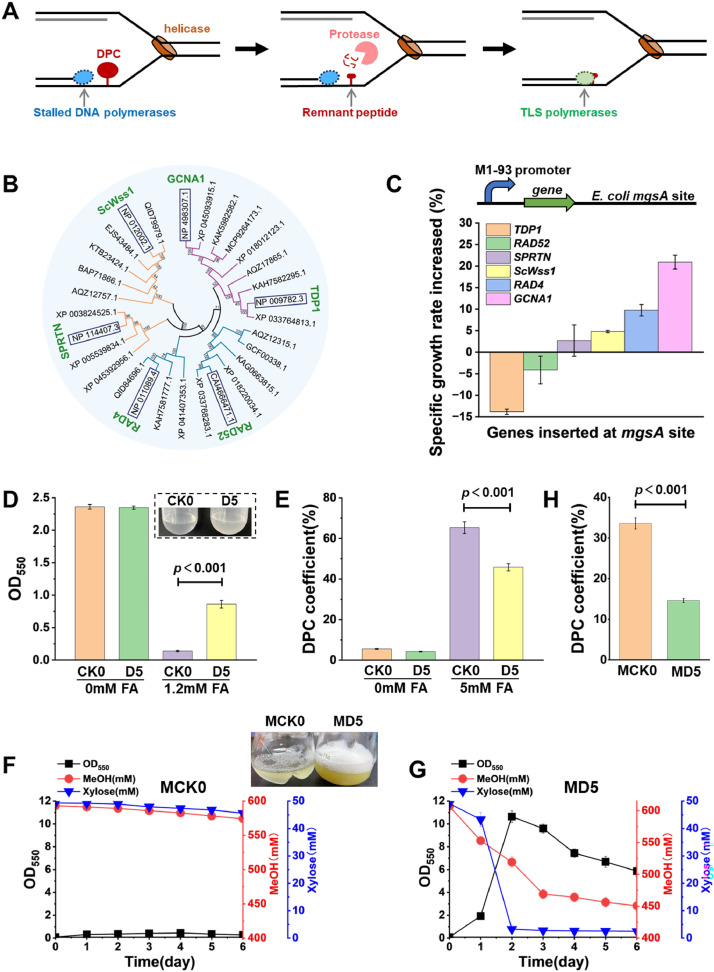
**Improving methanol assimilation by repairing formaldehyde-causing DPC.** (a) DPC proteases mitigate DPC damage through proteolysis. DPC located on the leading or lagging strand can impede the progress of DNA polymerase; proteolysis effectively degrades the majority of DPC, converting the crosslinked protein into residual peptides that are amenable to bypass by TLS (translesion synthesis) polymerase, thereby facilitating the continuation of DNA replication. (b) Molecular phylogenetic analysis of proteins involved in alleviating DPC damage. (c) The increased specific growth rate of strains was evaluated following the integration of genes at the *mgsA* site using 1 mM formaldehyde in MOPS medium supplemented with 2% (wt/v) glucose, incubated in a clear-bottom 96-well plate at 37 °C with an initial pH of 7.0. (d) OD_550_ values were measured for CK0 and D5 strains after a 35-hour incubation period with 0 mM or 1.2 mM formaldehyde in 10 mL MOPS medium supplemented with 2% (wt/v) glucose in 50 mL centrifugation tubes, maintained at 37 °C, 220 rpm, and an initial pH of 7.0. (e) Detection of DPC in CK0 and D5 strains after exposure to 0 mM or 5 mM formaldehyde for 10 h at 37 °C, 220 rpm, and an initial pH of 7.0. (f) & (g) Growth dynamics and metabolic utilization of methanol and xylose by both strains during cultivation in 40 mL MOPS medium containing 600 mM methanol and 50 mM xylose in 250 mL baffled flasks. (h) Detection of DPC coefficient in MCK0 and MD5 strains cultivated in LB medium with 600 mM methanol and 0.1 mM IPTG.

For efficient bio-assimilation of methanol, it is essential to effectively address the toxicity of formaldehyde. Among the toxicities caused by formaldehyde, DPC poses a significant threat primarily due to their bulky nature, which hinders the binding of polymerases and repair proteins to chromosomal DNA (Fig. 3a) [39,40]. To this end, we next endeavored to mitigate DPC. DPC can be removed through specific proteases that target the protein constituent of the DPC (DPC proteolysis). Nevertheless, there has been no report regarding the identification of an endogenous DPC protease in *E. coli*. Hence, we aimed to enlist a heterologous DPC protease from the enzyme database. Through a systematic molecular phylogenetic analysis, three DPC proteases, including SPRTN [41] from *Homo sapiens*, GCNA1 [42] from *Caenorhabditis elegans*, ScWss1 [43] from *Saccharomyces cerevisiae*. In addition, TDP1 [44], RAD4 [43] and RAD52 [43] those three proteins that may reduce DPC were also selected, all of which were derived from *Saccharomyces cerevisiae* (Figs. 3b and S2). The sequence identity of these six candidate proteins is only 7.9% (Fig. S3).

We then sought to introduce these candidate proteins into wild-type *E. coli* MG1655. As plasmid overexpression often causes obvious burdens to host cells, as well as additional and expensive inducers (e.g., IPTG, cumate) have to be added for turning on gene’s expression [45], we thus sought to integrate their encoding genes into the genomic DNA of *E. coli*, to express these genes in a plasmid- and inducer-free manner. To do this, a previously developed constitutive M1–93 promoter [28] was placed in front of each gene, and the constructed M1–93-*gene* expression cassette was inserted into the genome of MG1655 at the *mgsA* site (Fig. 3c), to yield the strains MG1655 *mgsA*::M1–93*-gene* (Table S3). The *mgsA* gene was also deleted to yield the control strain CK0 (MG1655 Δ*mgsA*).

We next assessed formaldehyde tolerance in these engineered strains at varying concentrations of formaldehyde by monitoring growth in MOPS + 2% glucose medium using clear-bottom 96-well plates with continuous shaking for 18 h at 37 °C and pH 7.0. In the absence of formaldehyde, both the control (CK0) and engineered strains grew normally without significant differences (Fig. S4). However, in the presence of 1 mM formaldehyde, the DPC-mitigating enzymes showed a distinct effect on formaldehyde tolerance of *E. coli.* Upon introduction of TDP1 and RAD52, the specific growth rates (μ, *h*^−1^) of *E. coli* strains exhibited reductions of 14% (0.234 h^−1^, *P* < 0.001) and 4% (0.261 h^−1^) (Fig. 3c), respectively, in comparison to the control strain (0.272 h^−1^). Conversely, the introduction of SPRTN showed minimal impact on growth rates under identical conditions. Intriguingly, introducing ScWss1, RAD4, and GCAN1 enhanced specific growth rates at 1 mM formaldehyde. Among them, the introduction of GCAN1 (termed strain D5) yielded the most pronounced effect, increasing the specific growth rate by 21% (0.328 h^−1^, *P* < 0.001) compared to the control strain CK0 (0.272 h^−1^). The soluble expression of GCNA1 in *E. coli* was also verified by SDS-PAGE (Fig. S5). We further validated the formaldehyde-resisting performance of strain D5 by transitioning it from the initial 96-well plate to a 50 mL centrifuge tube. Intriguingly, in the presence of 1.2 mM formaldehyde, the OD_550_ of the control CK0 strain only increased from 0.10 to 0.14 in up to 35 h, with an increase of 0.04 (Fig. 3d). In contrast, the OD_550_ of the D5 strain increased from 0.10 to 0.86 in the same time frame. The OD_550_ increase of the engineered strain is 19-fold (*P* < 0.001) higher than that of the control strain. Therefore, considering both the 96-well plate and the 50 mL centrifuge tube data, we can conclude that the D5 strain exhibits increased tolerance to formaldehyde.

Next, we verified whether the enhanced formaldehyde tolerance of the D5 strain stems from mitigating DPC in *E. coli* [46]. In the absence of formaldehyde, both CK0 and D5 strains exhibited comparable DPC coefficients, hovering around 5% (Fig. 3e). Following treatment with 5 mM formaldehyde, the DPC coefficient of the CK0 strain surged to 65%. In contrast, the DPC coefficient of the D5 strain stood at 46%, marking a notable 29% (*P* < 0.001) decline compared to its CK0 counterpart. This observation confirmed that the D5 strain does mitigate the formaldehyde-induced DPC, thereby bolstering its formaldehyde tolerance.

Since GCNA1 efficiently mitigated the DPC caused by formaldehyde, we next sought to harness it for methanol assimilation. We generated the related strain MD5 (MCK0, Δ*mgsA*::M1–93-*gcna1*) (Table S2). Intriguingly, under the same cultivation conditions as MCK0, the MD5 strain exhibited rapid growth, with cell mass reaching OD_550_ = 10.6 at 600 mM methanol, which was significantly higher by 29-fold (*P* < 0.001) compared to the MCK0 strain (OD_550_ = 0.36) (Fig. 3f, g). Additionally, it metabolized 146 mM methanol and 47 mM xylose within 6 days, marking a substantial 16- and 12-fold (*P* < 0.001) increase compared to the MCK0 strain (9 mM methanol, 4 mM xylose) (Fig. 3f, g). Consistent with the above result, the DPC coefficient in the MD5 strain was only 15%, representing a substantial 56% (*P* < 0.001) reduction compared to the MCK0 strain (34%) (Fig. 3h). These results highlight the crucial role of repairing formaldehyde-induced DNA damage in enhancing methanol assimilation.

### 3.3 Improving methanol assimilation by repairing formaldehyde-induced protein damage

**Fig. 4:**
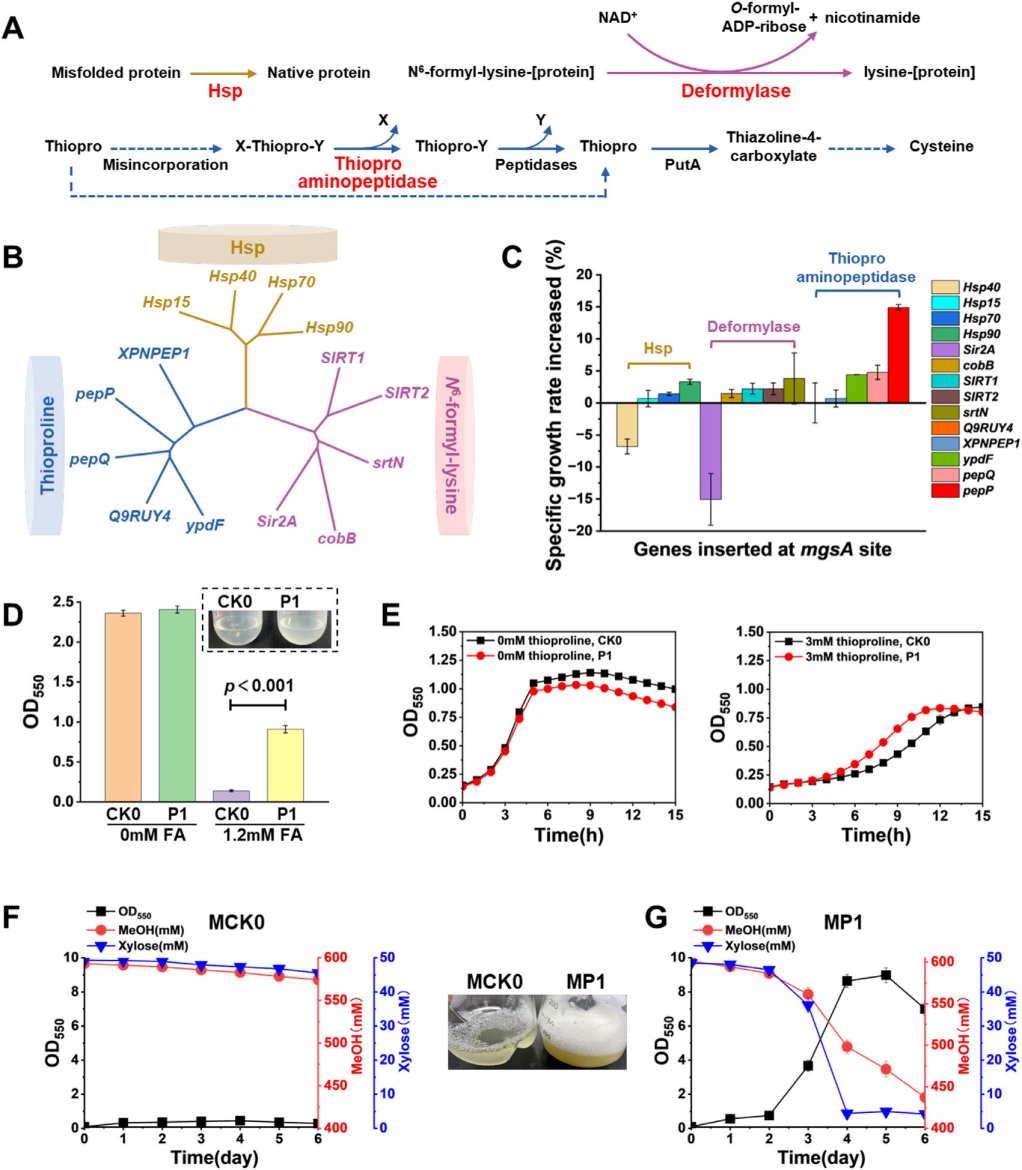
**Mitigating formaldehyde-generating protein damage and improving methanol assimilation.** (a) A schematic diagram illustrating the mechanism for detoxifying thioproline, *N*^6^-formyl-lysine and misfolded protein. Heat shock proteins are employed as the final approach to rectify misfolded proteins; Deformylase involves the deformylation of *N*^6^-formyl-lysine residues; Thiopro aminopeptidase can cleave thioproline-containing peptides, thereby facilitating the transformation towards cysteine. Thiopro: thioproline, X and Y represent any amino acid residues. (b) Three gene classes identified with potential for refolding misfolded proteins, detoxifying thioproline and *N*^6^-formyl-lysine. (c) The increased specific growth rate of strains with gene insertion at the *mgsA* site in the presence of 1 mM formaldehyde in MOPS + 2% (wt/v) glucose, assessed in a transparent-bottom 96-well plate at 37 °C with an initial pH of 7.0. (d) OD_550_ measurements of CK0 and P1 strains following 35 h of growth in 10 mL MOPS + 2% (wt/v) glucose with 0 mM or 1.2 mM formaldehyde, conducted in 50 mL centrifugation tubes at 37 °C, 220 rpm, and an initial pH of 7.0. (e) Growth performance of CK0 and P1 strains in the presence of 0 mM or 3 mM thioproline in MOPS + 2% (wt/v) glucose, analyzed in a clear-bottom 96-well plate at 37 °C with an initial pH of 7.0. (f) & (g) Growth dynamics and metabolic utilization of methanol and xylose by both strains during cultivation in 40 mL MOPS medium containing 600 mM methanol and 50 mM xylose in 250 mL baffled flasks.

Besides causing chromosomal DNA damage, formaldehyde has been reported to generate severe protein damage. Next, we aimed to repair formaldehyde-induced protein damage. After treatment with 10 mM formaldehyde for 10 h, we observed that the total intracellular soluble protein concentration of *E. coli* MG1655 decreased by 24% (*P* < 0.001) (Fig. S6). This result highlights the negative impact of formaldehyde on the misfolding and aggregation of intracellular proteins via causing protein cross-linking. Subsequently, we endeavored to address this issue. Heat shock proteins (Hsp) serve as molecular chaperones, supporting proper folding of proteins and assisting in the refolding of proteins that have been denatured by cellular stress [47] (Fig. 4a). Therefore, we sought to enhance the overexpression of *E. coli*’s endogenous HSPs to alleviate the formaldehyde-induced intracellular protein cross-linking, including Hsp15, Hsp40, Hsp70, and Hsp90 (Fig. 4b). The sequence identity of these HSPs is 8.5% (Fig. S7). Similarly, the constitutive M1–93 promoter was positioned upstream of each encoding gene, and the designed M1–93-*gene* expression cassette was integrated into the genome of MG1655 at the *mgsA* site, resulting in the generation of strains MG1655 Δ*mgsA*::M1–93-*Hsps* (Table S3). However, despite these efforts, the engineered strains did not exhibit notably enhanced formaldehyde tolerance (Figs. 4c and S8).

Besides causing protein cross-linking, we realized that formaldehyde can spontaneously react with amino and thiol groups of proteins, generating toxic compounds, e.g., thioproline and *N*^6^-formyl-lysine [48,49]. Therefore, we sought to mitigate the toxicity of these chemicals. Through a systematic molecular phylogenetic analysis, we selected 5 thioproline aminopeptidase involved in the detoxification of thioproline, including PepQ from *Pyrococcus furiosus*, XPNPEP1 from *Homo sapiens*, Q9RUY4 from *Deinococcus radiodurans*, PepP and YpdF from *E. coli* (Fig. S9). The sequence identity of these enzymes is 22.4% (Fig. S10). For the detoxification of *N*^6^-formyl-lysine, this selection comprises 5 deacetylases including SrtN from *Bacillus subtilis,* Sir2A from *Plasmodium falciparum,* CobB from *E. coli,* SIRT1 and SIRT2 from *H. sapiens* (Fig. S11). The sequence identity of these enzymes is 19.6% (Fig. S12).

Using the aforementioned genetic manipulation techniques, we integrated individual genes into the MG1655 genome, generating strains P1 through P10 (Table S3). We observed that these protein damage-mitigating enzymes showed a distinct effect on the formaldehyde tolerance of *E. coli.* Upon introduction of the Sir2A, the specific growth rate exhibited a 15% (*P* < 0.01) reduction compared to the control strain (CK0) when exposed to 1 mM formaldehyde (Figs. 4c and S13). Introduction of the remaining enzymes led to varying degrees of enhanced tolerance to formaldehyde. Notably, three candidates, namely YpdF, PepQ, and PepP, all classified under thioproline aminopeptidases, displayed the most significant improvement in formaldehyde tolerance (Figs. 4c and S14). Among them, PepP (MG1655 Δ*mgsA*::M1–93-*pepP*, P1) demonstrated the most prominent effect, with its specific growth rate increasing by 15% (*P* < 0.001) compared to the CK0 strain. To confirm the formaldehyde-resisting effect of strain P1, we further perform the formaldehyde tolerance assay in 50 mL centrifuge tubes. In the presence of ∼1.2 mM formaldehyde, the OD_550_ of the P1 strain increased from 0.10 to 0.91 in 35 h (Fig. 4d). The OD_550_ increase of the engineered strain is 20-fold higher than that of the control strain (∼0.04) (*P* < 0.001). These results confirmed that PepP did enhance formaldehyde tolerance and underscored the critical importance of fine-tuning gene expression in balancing damage repair while minimizing cellular burden.

To confirm that the enhanced formaldehyde tolerance of the P1 strain stems from detoxifying thioproline in *E. coli*, we performed the thioproline toxicity assay. Without thioproline, both CK0 and P1 exhibited similar growth rates. Upon the addition of 3 mM thioproline, P1 demonstrated significantly faster growth compared to CK0 (Figs. 4e and S15). The specific growth rate of the P1 strain (0.188 h^−1^) was 41% (*P* < 0.001) higher than that of CK0 (0.133 h^−1^). This observation suggests that incorporating the PepP can mitigate the toxicity of thioproline and thus enhance the formaldehyde tolerance.

To investigate the impact of *pepP* gene insertion on methanol assimilation, the *pepP* gene was subsequently integrated into the *mgsA* site of MCK0, yielding the MP1 strain. Under the same cultivation conditions as MCK0, the MP1 strain grew rapidly, with its biomass reaching an OD_550_ of 8.6 on day 4, representing a 24-fold (*P* < 0.001) increase over that of the MCK0 strain (OD_550_ = 0.36) (Fig. 4f, g). The MP1 strain also utilized 154 mM of methanol and 44 mM of xylose within 6 days, corresponding to 17- and 11-fold (*P* < 0.001) increases compared to the control strain MCK0 (9 mM methanol, 4 mM xylose). These results highlight the crucial role of repairing formaldehyde-induced protein damage in improving methanol assimilation.

### 3.4 Enhancing methanol assimilation by synergistic repair of DNA and protein damages

**Fig. 5:**
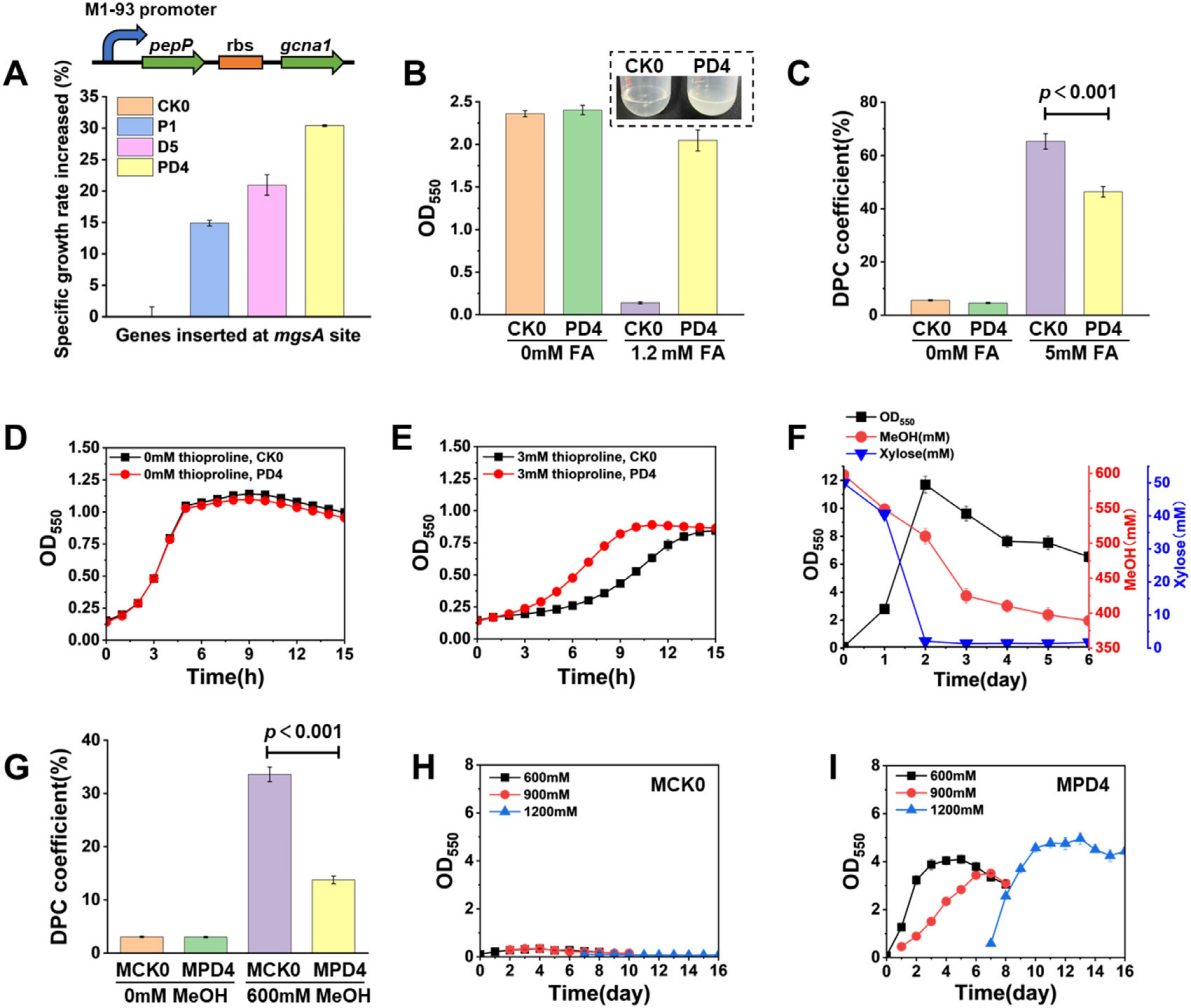
**Synergistic repair of DNA and protein damage further enhances methanol assimilation.** (a) The increase in specific growth rate of strains P1, D5 and PD4 was compared under 1 mM formaldehyde in MOPS + 2% (wt/v) glucose. Experiments were conducted in transparent-bottom 96-well plates at 37 °C and an initial pH of 7.0. (b) OD_550_ of CK0 and PD4 strains after 35 h of growth in the presence (1.2 mM) and absence of formaldehyde in 10 mL MOPS medium with 2% (wt/v) glucose in 50 mL centrifugation tubes, maintained at 37 °C, 220 rpm, with an initial pH of 7.0. (c) Detection of DPC in CK0 and PD4 strains following treatment with 5 mM formaldehyde for 10 h at 37 °C, 220 rpm, and an initial pH of 7.0. (d) & (e) Growth of PD4 strain in the presence (3 mM) and absence of thioproline in MOPS medium supplemented with 2% (wt/v) glucose in a clear-bottom 96-well plate incubated at 37 °C with an initial pH of 7.0. (f) Growth curve of MPD4 strain and the consumption of methanol and xylose during cultivation in 40 mL MOPS medium containing 600 mM methanol and 50 mM xylose in 250 mL baffled flasks. (g) Detection of DPC in MCK0 and MPD4 strains cultivated in LB medium with 0.1 mM IPTG, 0 mM or 600 mM methanol. (h) & (i) Growth curves of MCK0 or MPD4 strain cultivated in 10 mL MOPS medium supplemented with gradient concentrations of methanol and 50 mM xylose in 50 mL centrifugation tubes.

Since the repair of formaldehyde-induced DNA and protein damage individually mitigated formaldehyde toxicity, we wondered whether they could synergistically improve formaldehyde tolerance. To assess this, both GCNA1 and PepP encoding genes were co-inserted into the *mgsA* site, yielding strain PD4 (MG1655 Δ*mgsA*::M1–93-*pepP-gcna1*) (Fig. 5a). When cultivated in MOPS + 2% glucose medium with 1 mM formaldehyde in 96-well plates at 37 °C, the specific growth rate of PD4 strain was 30% (*P* < 0.001) higher than that of CK0 strain, which was twice and 1.4 times higher than that of P1 and D5, respectively (Figs. 5a and S16). Subsequently, the formaldehyde concentration was raised to 1.2 mM in 50 mL centrifuge tube. The OD_550_ of the PD4 strain increased from 0.10 to 2.1 in 35 h (Fig. 5b), which represents a 50-fold (*P* < 0.001) increase over that of the control strain (∼0.04). This high formaldehyde tolerance was significantly improved compared to *E. coli* that has been reported [48,50,51]. We further assessed mitigation of DNA and protein damage in the PD4 strain. Upon exposure to 5 mM formaldehyde, the DPC efficiency for the PD4 strain was only 47%, denoting a reduction of approximately 28% (*P* < 0.001) when compared with CK0 (∼65%) (Fig. 5c). In the presence of 3 mM thioproline, PD4 manifested a 63% (*P* < 0.001) increase in specific growth rate relative to CK0 (Figs. 5d, e, and S17). These findings suggest that the PD4 strain synergistically leverages the functionalities of the GCNA1 and PepP, efficiently mitigating DNA and protein damages.

We further evaluated the synergistic repair of DNA and protein damages on methanol assimilation by creating strain MPD4 (MCK0, Δ*mgsA*::M1–93-*pepP-gcna1*). These strains were then cultivated in shake flasks using MOPS minimal medium supplemented with 600 mM methanol, 50 mM xylose, and 2% (wt/v) casamino acids. Strain MPD4 grew to an OD_550_ of 12.0 on day 4, representing a 33-fold (*P* < 0.001) increase over that of the MCK0 strain (OD_550_ = 0.36). MPD4 exhibited robust consumption of 200 mM (∼6.7 g/L) methanol and 48 mM (∼7.2 g/L) xylose, showcasing a striking 22-fold and 13-fold (*P* < 0.001) increase, respectively, compared to the control strain MCK0 (9 mM methanol, 4 mM xylose) (Fig. 5f). The DPC coefficient in the MPD4 strain was only 14%, representing a substantial 59% reduction compared to the MCK0 strain (34%) (*P* < 0.001) (Fig. 5g).

We further investigated the growth and methanol assimilation capabilities of both strains at elevated methanol concentrations ranging from 900 to 1200 mM (Fig. 5h, i). Notably, the MPD4 strain demonstrated the ability to grow in the presence of 1200 mM methanol (∼38.4 g/L), whereas the growth of the MCK0 control strain was completely inhibited under these conditions (Fig. 5h, i). These findings underline the crucial role of repairing DNA and protein damages caused by formaldehyde in enhancing methanol assimilation.

### 3.5 Shifting the metabolism of *E. coli* from conventional sugar catabolism to requiring methanol as an essential substrate

**Fig. 6:**
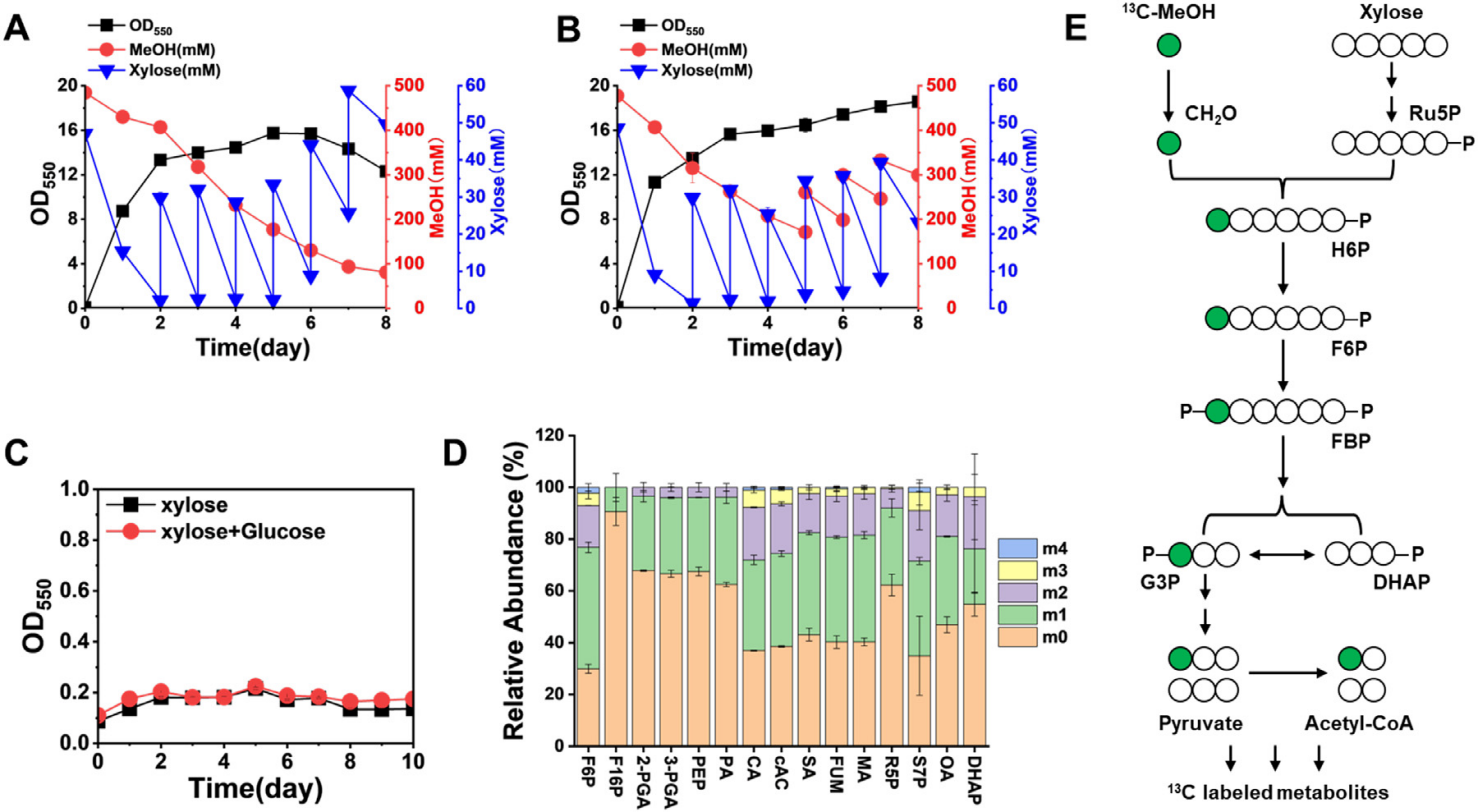
**The metabolic preference of the genetically engineered *E. coli* strains was altered.** (a) & (b) The growth curve and methanol, xylose consumptions of the MPD4 strain were investigated under the cultivation conditions of 40 mL MOPS medium containing 500 mM methanol and 50 mM xylose in 250 mL baffled flasks. Panel A represents the growth curve with intermittent xylose supplementation during fermentation, while Panel B illustrates the growth curve with additional methanol and xylose feeding throughout the fermentation process. (c) The growth curve of the MPD4 strain was examined in 40 mL MOPS medium devoid of methanol. (d) The labeling abundance of intracellular metabolites originating from ^13^C-methanol was quantified, including F6P, fructose 6-phosphate; F16P, fructose 1;6-diphosphate; 2-PGA, 2-phosphoglyceric acid; 3-PGA, 3-phosphoglyceric acid; PEP, phosphoenolpyruvic acid; PA, pyruvic acid; CA, citric acid; cAC, cis-Aconitic acid; SA, succinic acid; FUM, fumaric acid; MA, malic acid; R5P, ribose-5-phosphate; S7P, sedoheptulose 7-phosphate; OA, oxoglutaric acid; DHAP, dihydroxyacetone phosphate. (E) Carbon labeling of metabolites derived from ^13^C-methanol with co-utilization of xylose in the engineered *E. coli* strains.

We subsequently optimized the cultivation conditions of strain MPD4 in baffled flasks, further enhancing methanol assimilation. When solely fed with xylose during fermentation, the MPD4 strain reached an OD_550_ = 16 at day 6, and consumed 388 mM methanol (∼12.4 g/L) within 8 days (Fig. 6a). Upon completion of an 8-day fermentation period, it was noted that the methanol concentration in the medium had diminished to below 100 mM. Consequently, in subsequent experiments, we initiated xylose supplementation from the 5th day onwards. Upon continuous feeding of methanol and xylose during fermentation, the MPD4 strain exhibited rapid growth, attaining its peak biomass at OD_550_ = 19 by consuming 440 mM methanol (∼14.1 g/L) within 8 days (Fig. 6b), which represents 52- and 50-fold (*P* < 0.001) increases, respectively, compared to the control strain (OD_550_ = 0.36; 9 mM methanol). Furthermore, through eight rounds of passage tests, we have also proved that the passage growth remains stable (Fig. S18).

Notably, the MPD4 strain failed to proliferate in MOPS minimal medium devoid of methanol (0 mM), supplemented with 50 mM xylose and 2% (wt/v) casamino acids (Fig. 6c). Even the inclusion of 2% (wt/v) glucose failed to stimulate its growth (Fig. 6c). Conversely, robust growth was observed in the presence of methanol, signifying a shift from conventional sugar metabolism to a requisite for methanol as a growth-supporting substrate.

To verify this, we conducted the ^13^C-methanol-labeling analysis of MPD4 in a MOPS medium containing 50 mM xylose and 100 mM ^13^C-methanol. Cells were harvested after approximately 8 days, when most methanol had been utilized. The analysis detected key metabolites involved in carbon metabolism, including intermediates associated with the Embden-Meyerhof-Parnas (EMP) pathway, tricarboxylic acid (TCA) cycle, and pentose phosphate pathway (PPP) [19]. Following an 8-day cultivation period, significant portions of labeled carbon derived from methanol were observed in various metabolites within the EMP pathway, such as 70% fructose 6-phosphate, 9% fructose 1,6-diphosphate, 32% 2-phosphoglyceric acid, 33% 3-phosphoglyceric acid, 32% phosphoenolpyruvic acid, 37% pyruvic acid, and 45% dihydroxyacetone phosphate (Fig. 6d). Furthermore, substantial labeling was found in key compounds of the TCA cycle, with 63% citric acid, 61% cis-aconitic acid, 57% succinic acid, 60% fumaric acid, 60% malic acid, and 53% oxoglutaric acid showing labeled carbon. Additionally, 38% ribose-5-phosphate and 65% sedoheptulose 7-phosphate within the PPP exhibited labeling. Furthermore, a significant proportion of the detected metabolites exhibited labeling with multiple carbons. Within the metabolites associated with central carbon metabolism, approximately 20% of citric acid and dihydroxyacetone phosphate, 19% of cis-aconitic acid and sedoheptulose 7-phosphate, and around 16% of fructose 6-phosphate, succinic acid, fumaric acid, malic acid, and oxoglutaric acid displayed *M* + 2 labeling (Fig. 6d). Additionally, > 5% of citric acid, cis-aconitic acid, and sedoheptulose 7-phosphate showcased *M* + 3 labeling. Moreover, roughly 2% of fructose 6-phosphate and sedoheptulose 7-phosphate exhibited *M* + 4 labeling. These findings collectively indicate that methanol is effectively integrated into the central carbon metabolism of the MPD4 strain (Fig. 6e).

### 3.6 Production of valuable products from methanol using strain MPD4

**Fig. 7:**
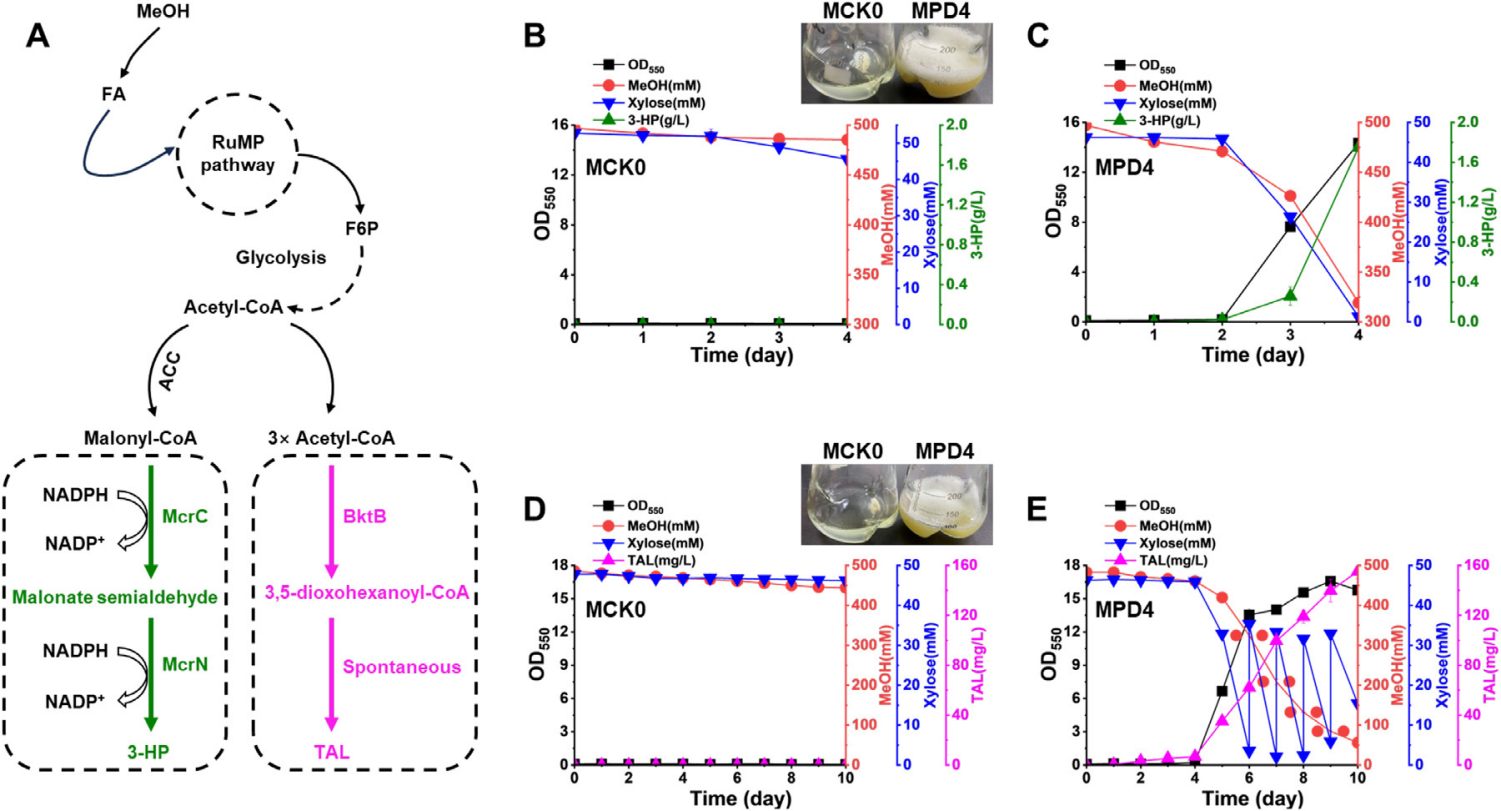
**The utility of methanol-assimilating *E. coli* to produce 3-HP and TAL.** (A) The metabolic pathways to produce 3-HP and TAL. Methanol is incorporated into fructose 6-phosphate through the RuMP pathway, which then forms acetyl-CoA. Acetyl-CoA is then converted into malonyl-CoA via acetyl-CoA carboxylase (ACC), and further catalyzed by McrC and McrN to produce 3-HP. Three molecules of acetyl-CoA are catalyzed by BktB to form 3,5-dioxohexanoyl-CoA, which subsequently undergoes a spontaneous reaction to yield TAL. (B) & (C) Growth, methanol and xylose consumption, and 3-HP production for strains MCK0+pET-3-HP (left) and MPD4+pET-3-HP (right). (D) & (E) Growth, methanol and xylose consumption, and TAL production for strains MCK0+pTrc99a-TAL (left) and MPD4+pTrc99a-TAL (right). Error bars represent the standard error of the mean (*n*  =  3).

We aimed to harness the engineered strain MPD4 for synthesizing valuable bioproducts using methanol as a substrate. One such compound of interest is 3-hydroxypropionate (3-HP), which acts as a crucial precursor for a variety of chemicals, including acrylate and acrylamide, and serves as a monomer in the production of biodegradable plastics [52]. As a notable recognition of its significance, 3-HP has been identified as one of the top 12 biobased building blocks by the United States Department of Energy (DOE) [53]. In our endeavor, we employed the engineered *E. coli* strains for bioproduction of 3-HP. Given that 3-HP is not naturally produced by *E. coli*, we initially engineered a heterologous biosynthetic pathway for 3-HP, utilizing malonyl-CoA as a precursor (Fig. 7a). Specifically, we introduced the *mcrN* and *mcrC* genes encoding malonyl-CoA reductase from *Chloroflexus aurantiacus* [54]. Subsequently, the resultant plasmid pET-*mcrN*-*mcrC* was transformed into strains MCK0 and MPD4.

The MCK0 strain, carrying pET-*mcrN*-*mcrC*, exhibited a lack of growth when cultivated in baffled flasks using MOPS minimal medium supplemented with 500∼600 mM methanol, 50 mM xylose, and 2% (wt/v) casamino acids. This strain displayed minimal assimilation of methanol and xylose, and no detectable production of 3-HP (Fig. 7b). In contrast, the MPD4 strain, also harboring pET-*mcrN*-*mcrC*, demonstrated the ability to utilize up to 171 mM methanol (∼5.5 g/L) and 45 mM xylose, resulting in the production of approximately 190 mM (∼1.7 g/L) of 3-HP within a 4-day timeframe (Fig. 7c). Notably, the 3-HP production yield achieved by the MPD4 strain surpassed that of previously reported bacterial fermentations utilizing methanol substrate [[55], [56], [57]].

In addition to 3-HP, triacetate lactone (TAL) represents another versatile platform chemical with applications in synthesizing various molecules [58]. In this study, we leveraged the MPD4 strain for TAL production by utilizing the previously developed plasmid pTrc99a-*bktB* (Fig. 7A). Strain MPD4 carrying the pTrc99a-*bktB* plasmid exhibited TAL production at a level of approximately 155 ± 2 mg/L within a 10-day period, while the control strain was unable to grow, consume methanol or produce TAL under the same conditions (Fig. 7d, e). Collectively, these findings underscore the efficacy of the engineered *E. coli* strain MPD4 as a robust chassis for the biosynthesis of valuable compounds from methanol.

## 4 Discussion

Methanol is now considered as a next-generation substrate for the biomanufacturing industry [59]. In yeast cells, the implementation of various strategies, such as reducing methanol toxicity, enhancing precursor donor supply, and promoting cofactor regeneration, can markedly improve the assimilation efficiency of methanol [2,60]. However, in *E. coli*, the ability to utilize methanol remains limited, and its assimilation is often impeded by formaldehyde cytotoxicity. Without addressing the cytotoxicity of formaldehyde, traditional rational engineering without evolution has only achieved methanol assimilation levels within the 10–150 mM range, with assimilation rates varying from 0.1 to 0.8 mM/h in all methanol-metabolizing *E. coli* strains (Table S1). In nature, microbes have evolved formaldehyde dissimilation pathways to detoxify formaldehyde into formate and eventually CO_2_ [18]. However, these pathways may not be directly utilized for formaldehyde detoxification during methanol assimilation due to the significant loss of the formaldehyde intermediate [5].

Adaptive laboratory evolution (ALE) may show promise in mitigating formaldehyde toxicity through accumulating beneficial mutations. Recent studies have successfully employed ALE strategy to obtain strains of *E. coli* that exhibit enhanced growth characteristics [61,62]. However, ALE frequently requires long timescales, on the order of months, and often necessitates daily attention. Further, these beneficial mutations are prone to re-installing formaldehyde dissimilation pathways, leading to re-wastage of formaldehyde [18]. In contrast, the strategy of rational repairing DNA and protein damages offers a novel and rapid approach to both mitigate formaldehyde toxicity and prevent carbon losses. Notably, all genetic manipulations involved can be completed within 1 day.

The MPD4 strain, derived from the synergistic repair of DPC and protein damage, exhibited exceptional performance. Specifically, it achieved methanol assimilation up to 440 mM at a rate of 2.3 mM/h. This represents a remarkable 3- to 44- fold enhancement in methanol consumption capacity and a 3- to 23- fold improvement in consumption rate compared to what is attainable through conventional rational engineering approaches (Table S1). In addition, the MPD4 strain exhibited growth capability in the presence of 1200 mM methanol (∼38.4 g/L). To our knowledge, this substrate concentration surpasses the substrate utilization levels observed in the majority of native methylotrophic bacteria, such as *B. methanolicus* [63] and *M. extorquens* [64] and non-native methylotrophic bacteria such as *E. coli* [25,65] and *C. glutamicum* [66,67], which typically utilize methanol at concentrations lower than 250 mM (∼8 g/L) [5].

In our search for enzymes involved in the repair of DNA and protein damage, we observed that PepP overexpression was reported not to enhance formaldehyde tolerance in wild-type *E. coli* [48], which seems contradictory to our results. We deem that this discrepancy may arise from their use of plasmid-based overexpression [48]. In contrast, we integrated *pepP* gene with a constitutive M1–93 promoter into the genomic DNA of *E. coli*, enabling gene expression in a plasmid-free manner, which efficiently alleviate cellular burden [45]. We also experimentally confirmed that some enzymes exhibit varying effects on the formaldehyde tolerance of *E. coli*. In particular, the introduction or overexpression of TDP1, RAD52, Hsp40, and Sir2A in *E. coli* had a detrimental impact on formaldehyde resistance. The specific reasons underlying these observations remain elusive and warrant further investigation in subsequent studies. These findings emphasize the significance of a systematic exploration and in-depth analysis to identify optimal enzymes for damage repair mechanisms.

Additionally, in this study, most of the methanol assimilation occurred in shake flask cultivations. Further refinement of cultivation conditions, which may involve adjusting working volumes, optimizing inducer concentrations, and adopting fed-batch fermentation with meticulous control over parameters like pH and dissolved oxygen levels [68], could lead to a further increase in methanol consumption. In this study, we successfully produced 3-HP at the gram scale using methanol as the substrate. Furthermore, our approach holds significant potential for expanding the range of methanol-derived products. Beyond the existing product range, this could encompass additional categories such as organic acids, organic bases, antibiotics, flavor compounds, and potentially many other high-value chemicals.

In this study, we selected methanol and the central RuMP pathway to demonstrate the effectiveness of repairing formaldehyde-causing damages in improving methanol assimilation. In principle, this strategy can also harness other methylotrophic pathways that use formaldehyde as an intermediate, including but not limited to XuMP pathway [20], homoserine cycle [21], MCC pathway [10], FLS pathway [15], SACA pathway [13], ASAP pathway [11], HACL pathway [16], and FORCE pathway [12,16] (Fig. 1). Besides methanol, this strategy can also be applied for efficient assimilation of other one-carbon feedstocks such as formate, methane, CO_2_, and CO. Furthermore, besides *E. coli*, the strategy can also be extended to any other microbe for methanol assimilation.

## 5 Conclusion

We utilized rational metabolic engineering approaches to bolster the formaldehyde tolerance of *E. coli*, effectively mitigating damages to DPC and proteins, which in turn significantly improved methanol assimilation. Methanol was effectively incorporated into the central carbon metabolism of the MPD4 strain, yielding a methanol utilization rate of 2.29 mM/h—nearly 50-fold higher than that of the control strain. Ultimately, the MPD4 strain demonstrated its ability to synthesize high-value chemicals. Our findings highlight the critical role of alleviating formaldehyde toxicity in methanol assimilation and offer new perspectives and strategies for enhancing the exploitation of one-carbon compounds.

## CRediT authorship contribution statement

**Cheng Zhu:** Writing – original draft, Methodology, Investigation. **Yun Chen:** Methodology, Investigation. **Wenjie Sun:** Methodology, Investigation. **Jian Li:** Methodology, Investigation. **Haiyan Liu:** Methodology, Investigation. **Jiahui Peng:** Methodology, Investigation. **Yanfen Bai:** Methodology, Investigation. **Ramon Gonzalez:** Writing – original draft, Resources, Methodology. **Zaigao Tan:** Writing – review & editing, Writing – original draft, Supervision, Project administration, Methodology, Investigation, Funding acquisition, Conceptualization.

## Declaration of competing interest

The authors declare that they have no conflicts of interest in this work.

## Appendix Supplementary materials

Image, application 1
