# Supplementary material for: Repair of DNA and protein damages caused by formaldehyde improves methanol assimilation
Source: Fundam Res. 2025 May 17;6(4):2331–42. doi: 10.1016/j.fmre.2025.05.007 (PMC13424408; doi:10.1016/j.fmre.2025.05.007)
Supplement: Supplementary file 1 [file mmc1.docx]

**Repair of DNA and protein damages caused by formaldehyde improves methanol assimilation**

Cheng Zhu^1,2^, Yun Chen^1,2^, Wenjie Sun^1,2^, Jian Li^1,2^, Haiyan Liu^1,2^, Jiahui Peng^1,2^,

Yanfen Bai^3^, Ramon Gonzalez^4^, and Zaigao Tan^1,2*^

^1^ State Key Laboratory of Microbial Metabolism, Shanghai Jiao Tong University, Shanghai, China. 200240.

^2^ Department of Bioengineering, School of Life Sciences and Biotechnology, Shanghai Jiao Tong University, Shanghai, China. 200240.

^3^ Shenzhen Institute of Advanced Technology, Chinese Academy of Sciences, Shenzhen, China. 518000.

^4^ Department of Chemical, Biological and Materials Engineering, University of South Florida, Tampa, FL, USA. 33620.

Cheng Zhu: [zhuchengshjd@sjtu.edu.cn](mailto:zhuchengshjd@sjtu.edu.cn)

Yun Chen: [chenyun0402ye@163.com](mailto:chenyun0402ye@163.com)

Wenjie Sun: [sunwenjie_007@163.com](mailto:sunwenjie_007@163.com)

Jian Li: [lij0813@163.com](file:///E:\Grade5\陈云\lij0813@163.com)

Haiyan Liu: [yuxihy55@163.com](file:///E:\Grade5\陈云\yuxihy55@163.com)

Jiahui Peng: 2224926970@sjtu.edu.cn

Yanfen Bai: yf.bai@siat.ac.cn

Ramon Gonzalez: [ramongonzale@usf.edu](mailto:ramongonzale@usf.edu)

*Corresponding author: Zaigao Tan, 224 Wenxuan Building, Shanghai Jiao Tong University, 800 Dongchuan Road, Minhang District, Shanghai, China, 200240, [ZTAN0918@sjtu.edu.cn](mailto:ZTAN0918@sjtu.edu.cn), +86-18221961734, ORCID ID: 0000-0003-4528-0723

### Supplementary Table 1. Representative progress in the rational design of methanol-assimilating Escherichia coli strains without adaptive laboratory evolution

| **Strain** | **Genetic modification** | **Medium component** | **Methanol consumption (mM)** | **Methanol consumption rate (mM/h)** | **Ref** |
| --- | --- | --- | --- | --- | --- |
| BW25113 | Knockout *frmA*  Expression of *mdh* from *B. stearothermophilus*, *hps* and *phi* from *B. methanolicus* | M9 minimal medium, supplemented with 60 mM methanol and 1 g/L yeast extract | 10 | 0.14 | [1] |
| W1485 | Knockout *pflAB*, *ldhA*, *ptsG*  Genes *mdh, hps and phi from B. methanolicus* and codon optimized for expression | CD medium, supplemented with 30 g/L glucose, 1 g/L yeast extract and 6.4 g/L methanol | 14 | 0.13 | [2] |
| BW25113 | Knockout *frmA*  Expression of *mdh2* from *B. methanolicus* and artificial FLS | M9 minimal medium, supplemented with 1 g/L yeast extract and 250 mM methanol | 14 | 0.44 | [3] |
| BW25113 | Knockout *frmA*  Expression of *mdh3* from *B. methanolicus*, *hps* and *phi* from *M. gastri* | M9 minimal medium, supplemented with 500 mM methanol | 18 | 0.73 | [4] |
| BL21(DE3) | Knockout *rpe*  Expression of *mdh2* from *C. necato*, *hps* from *B. methanolicus*, *phi* from *M. flagellates* | MOPS minimal medium, supplemented with 50 mM ribose, 250 mM methanol and 0.05% casamino acids | 21 | 0.44 | [5] |
| Suc260 | Expression of *mdh2* from *B. methanolicus*, *fdh* from *C. boidinii*, *pyc* from *L. lactis* | M9 medium, supplemented with 50 g/L glucose, 3.0 g/L sodium formate and 200 mM methanol | 31 | 0.3 | [6] |
| MG1655 | Knockout *frmAB*, *pfkAB, sucA*  Expression of the *mdh* from *A. garner*, *das* from *P. angusta*, and introducing the antisense RNA to inhibit the expression of *gapA* gene | M9 minimal medium with 1 g/L yeast extract and 400 mM methanol | 32 | 0.66 | [7] |
| BW25113 | Knockout *frmA*, *pgi*  Expression of *mdh* from *B. stearothermophilus, hps* and *phi* from *B. methanolicus*  Expressing the non-oxidative pentose phosphate pathway from *B. methanolicus* | M9 minimal medium, supplemented with 10 g/L yeast extract and 120 mM methanol | 38 | 0.79 | [8] |
| MG1655  (DE3) | Knockout *IdhA*, *frmA*, *rpiA*, *rpiB*, *cyaA*  Expression of *mdh* from *C. necator, hps* and *phi* from *B. methanolicus*  Expression of pmtA from *R. sphaeroides* | MOPS minimal medium, supplemented with 1% (wt/v) casamino acids, 50 mM xylose and 300 mM methanol | 146 | 0.61 | [9] |
| MCK0 | Knockout *mgsA*, *frmA*, *rpiA*, *rpiB*, *cyaA*  Expression of *mdh* from *C. necator, hps* and *phi* from *B. methanolicus* | MOPS minimal medium, supplemented with 2% (wt/v) casamino acids, 50 mM xylose and 600 mM methanol | 9 | 0.047 | This study |
| MPD4 | Knockout *mgsA*, *frmA*, *rpiA*, *rpiB*, *cyaA*  Expression of *mdh* from *C. necator, hps* and *phi* from *B. methanolicus*  Expression of *gcna1* from *C. elegans* and *pepP* from *E. coli* | MOPS minimal medium, supplemented with 2% (wt/v) casamino acids, 50 mM xylose and 500 mM methanol | 440 | 2.29 | This study |

Mdh: methanol dehydrogenase; Hps: 3-hexulose-6-phosphate synthase; Phi: 6-phospho-3-hexuloisomerase; FrmA: S-(hydroxymethyl) glutathione dehydrogenase; RpiAB: ribose-5-phosphate isomerase A and B; CyaA: adenylate cyclase; Pgi: phosphoglucose isomerase; Rpe: ribulose-5-phosphate 3-epimerase; LdhA: D-lactate dehydrogenase. PtsG: glucose-specific PTS enzyme IIBC component; PflAB: pyruvate formate-lyase.

### Supplementary Table 2. Strains utillized for methanol assimilation assays in the study.

| Strains | Description | Source |
| --- | --- | --- |
| MG1655(DE3) | MG1655 with DE3 integration | Lab collection |
| MCKE | MG1655(DE3) Δ*mgsA* Δ*frmA* Δ*rpiA* Δ*rpiB* Δ*cyaA*, containing plasmid pCDF-empty | This study |
| MCK0 | MG1655(DE3) Δ*mgsA* Δ*frmA* Δ*rpiA* Δ*rpiB* Δ*cyaA*, containing plasmid pCDF-RuMP | This study |
| MD5 | MG1655(DE3) Δ*mgsA*::M1-93-*gcna1* Δ*frmA* Δ*rpiA* Δ*rpiB* Δ*cyaA*, containing plasmid pCDF-RuMP | This study |
| MP1 | MG1655(DE3) Δ*mgsA*::M1-93-*pepP* Δ*frmA* Δ*rpiA* Δ*rpiB* Δ*cyaA*, containing plasmid pCDF-RuMP | This study |
| MPD4 | MG1655(DE3) Δ*mgsA*::M1-93-*pepP-RBS4*-*gcna1* Δ*frmA* Δ*rpiA* Δ*rpiB* Δ*cyaA*, containing plasmid pCDF-RuMP | This study |

### Supplementary Table 3. Strains for tolerance testing in this study.

| Strains | Description | Source |
| --- | --- | --- |
| MG1655 | Wild-type *E. coli* K-12 strain | Lab collection |
| CK0 | MG1655 Δ*mgsA* | This study |
| D1 | MG1655 Δ*mgsA*::M1-93-*ScWss1* | This study |
| D2 | MG1655 Δ*mgsA*::M1-93-*RAD4* | This study |
| D3 | MG1655 Δ*mgsA*::M1-93-*RAD52* | This study |
| D4 | MG1655 Δ*mgsA*::M1-93-*SPRTN* | This study |
| D5 | MG1655 Δ*mgsA*::M1-93-*GCNA1* | This study |
| D6 | MG1655 Δ*mgsA*::M1-93-*TDP1* | This study |
| H1 | MG1655 Δ*mgsA*::M1-93-*Hsp15* | This study |
| H2 | MG1655 Δ*mgsA*::M1-93-*Hsp40* | This study |
| H3 | MG1655 Δ*mgsA*::M1-93-*Hsp70* | This study |
| H4 | MG1655 Δ*mgsA*::M1-93-*Hsp90* | This study |
| P1 | MG1655 Δ*mgsA*::M1-93-*pepP* | This study |
| P2 | MG1655 Δ*mgsA*::M1-93-*pepQ* | This study |
| P3 | MG1655 Δ*mgsA*::M1-93-*XPNPEP1* | This study |
| P4 | MG1655 Δ*mgsA*::M1-93-*ypdF* | This study |
| P5 | MG1655 Δ*mgsA*::M1-93-*Q9RUY4* | This study |
| P6 | MG1655 Δ*mgsA*::M1-93-*srtN* | This study |
| P7 | MG1655 Δ*mgsA*::M1-93-*Sir2A* | This study |
| P8 | MG1655 Δ*mgsA*::M1-93-*cobB* | This study |
| P9 | MG1655 Δ*mgsA*::M1-93-*SIRT1* | This study |
| P10 | MG1655 Δ*mgsA*::M1-93-*SIRT2* | This study |
| PD4 | MG1655 Δ*mgsA*::M1-93-*pepP-RBS4*-*gcna1* | This study |

### Supplementary Table 4. Plasmids utillized in the study.

| Plasmids | Description | Source |
| --- | --- | --- |
| pCDF-duet | pCDF-duet empty plasmid, Spe^r^ | Lab collection |
| pET-duet | pET-duet empty plasmid, Amp^r^ | Lab collection |
| pTrc99a | pTrc99a empty plasmid, Amp^r^ | Lab collection |
| pCDF-RuMP | pCDF-duet carrying *mdh*, *hps* and *phi* for methanol assimilation, Spe^r^ | This study |
| pET-*mcrN-mcrC* | pET-duet carrying *mcrC* and *mcrN* from *Chloroflexus aurantiacus* for 3-HP production, Amp^r^ | This study |
| pTrc99a-*bktB* | pTrc99a carrying *bktB* from *Ralstonia eutropha* for TAL production, Amp^r^ | This study |

### Supplementary Table 5. Proteins used to alleviate DPC damage.

| Name | Amino acids | Uniprot ID |
| --- | --- | --- |
| TDP1 | 544 | P38319 |
| RAD52 | 471 | P06778 |
| SPRTN | 489 | Q9H040 |
| ScWss1 | 269 | P38838 |
| RAD4 | 754 | P14736 |
| GCNA1 | 532 | Q23462 |

### Supplementary Table 6. Proteins used to alleviate protein damage.

| Name | Amino acids | Uniprot ID |
| --- | --- | --- |
| Hsp15 | 133 | P0ACG8 |
| Hsp40 | 376 | P08622 |
| Hsp70 | 638 | P0A6Y8 |
| Hsp90 | 624 | P0A6Z3 |
| Sir2A | 273 | Q8IE47 |
| CobB | 279 | P75960 |
| SIRT1 | 747 | Q96EB6 |
| SIRT2 | 389 | Q8IXJ6 |
| SrtN | 247 | A0A6M4JHV4 |
| Q9RUY4 | 349 | Q9RUY4 |
| XPNPEP1 | 623 | Q9NQW7 |
| YpdF | 361 | P76524 |
| PepQ | 348 | P81535 |
| PepP | 441 | P15034 |


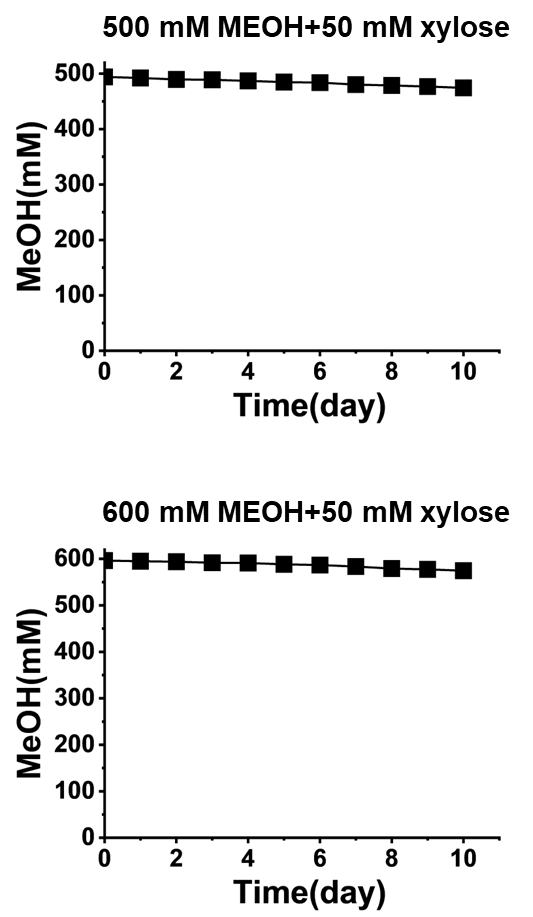


### Supplementary Figure 1. Determination of the methanol evaporation control. To minimize error, this set of experiments were performed in a 250ml baffle flask. Error bars indicate standard error of the mean (n = 3).


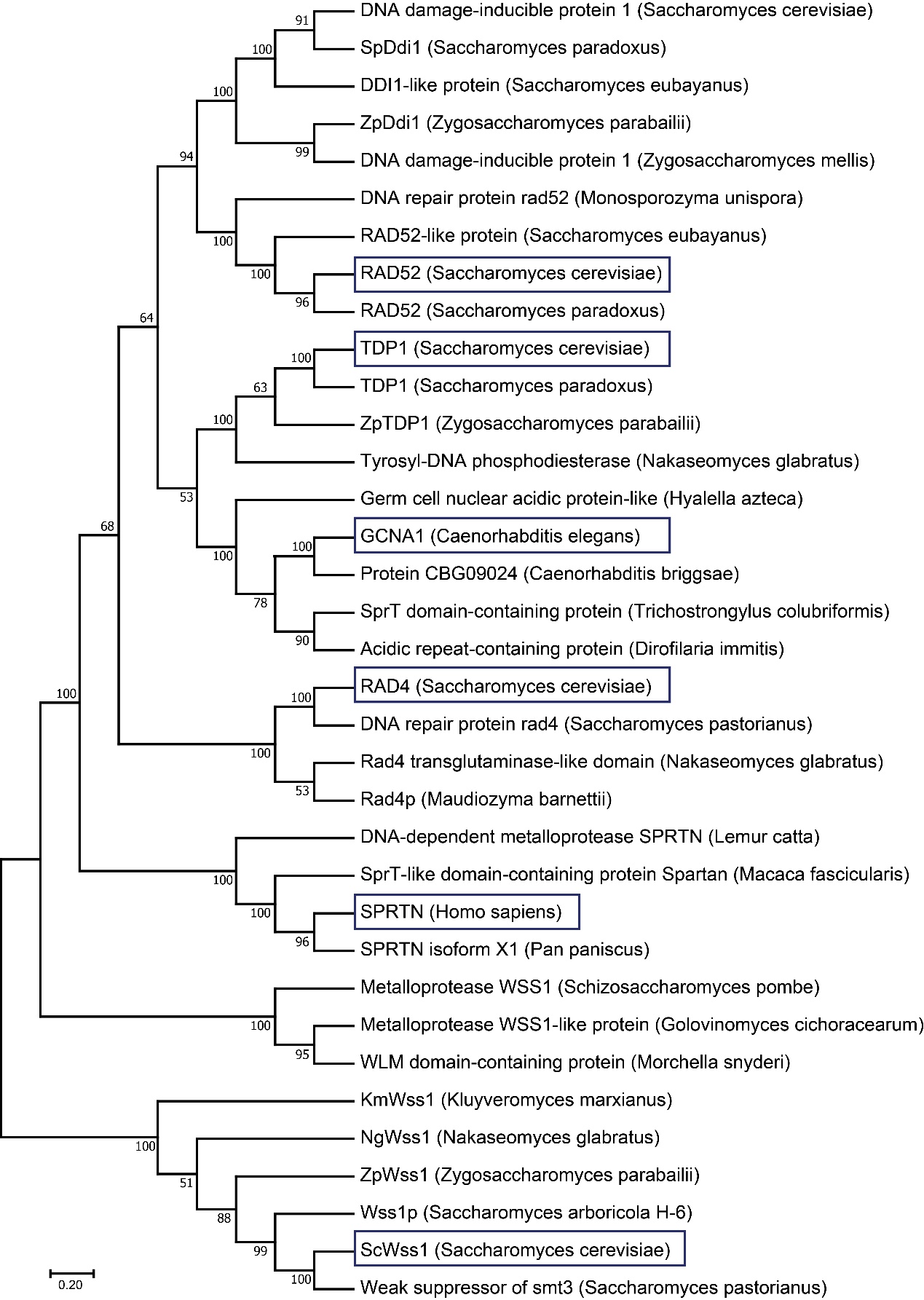


### Supplementary Figure **2. Molecular phylogenetic analysis of proteins involved in alleviating DPC damage.**


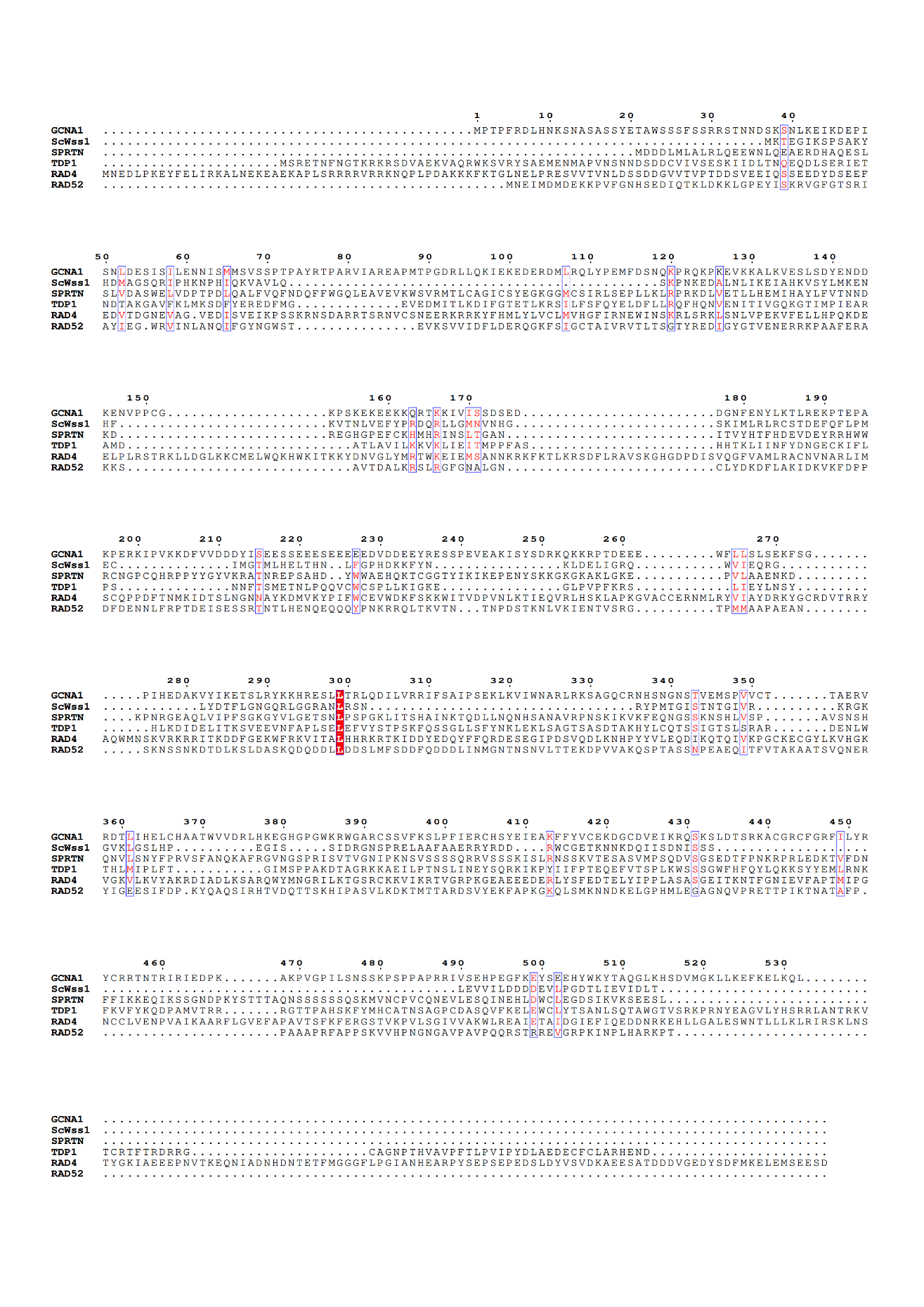


### Supplementary Figure 3. **Primary sequence alignment of proteins involved in alleviating DPC damage.** SPRTN from *Homo sapiens*, GCNA1 from *Caenorhabditis elegans*, ScWss1, TDP1, RAD4 and RAD52 from *Saccharomyces cerevisiae*. The sequence identity is 7.9%.


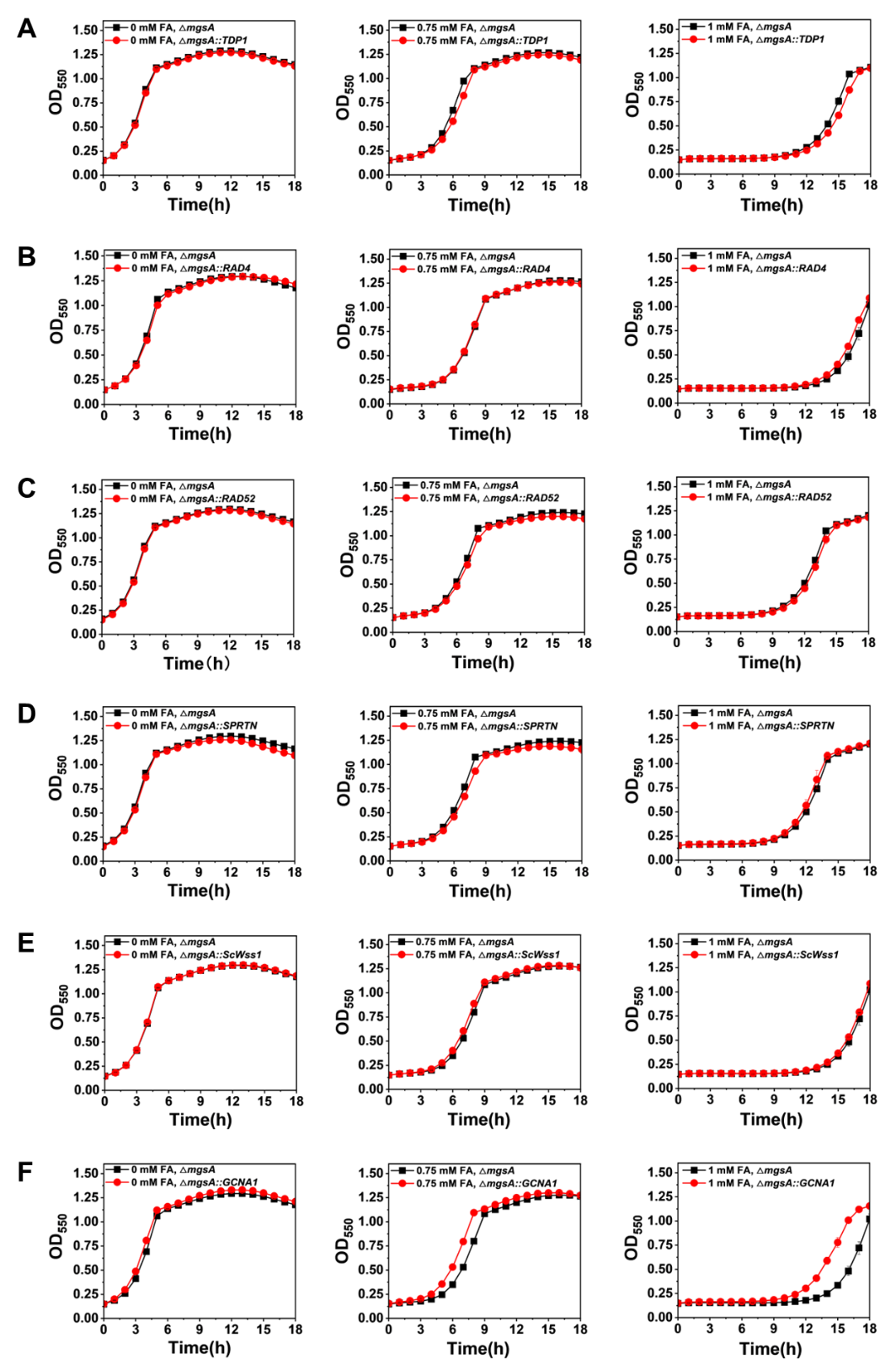


### Supplementary Figure 4. Formaldehyde tolerance of engineered strains utilized for mitigating DPC damage. (A) – (F) Genes *TDP1, RAD4, RAD52, SPRTN, ScWss1* and *GCNA1* were inserted into the *mgsA* site. The strains were cultured in the presence of 0 mM, 0.75 mM, and 1 mM formaldehyde in MOPS supplemented with 2% (wt/v) glucose in a clear-bottom 96-well plate at 37℃ and an initial pH of 7.0. Values are average of at least three biological replicates, and error bars indicate standard deviation.


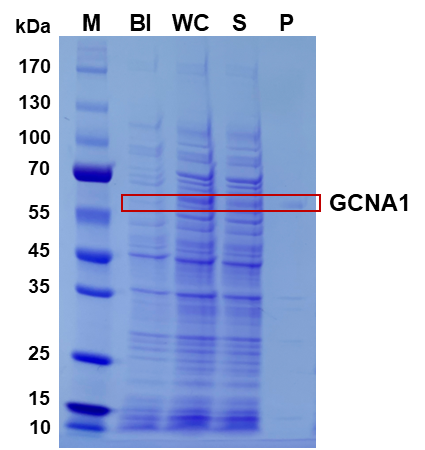


### Supplementary Figure **5. SDS-PAGE analysis of GCNA1 protein expression in *E. coli* MG1655.** Lane “M” represents the protein molecular weight marker, “BI” represents the sample before induction, “WC” indicates the whole cell sample following induction, “S” and “P” correspond to the supernatant and pellet fractions of the cell lysate, respectively. The theoretical molecular weight of the GCNA1 protein is 63 kDa.


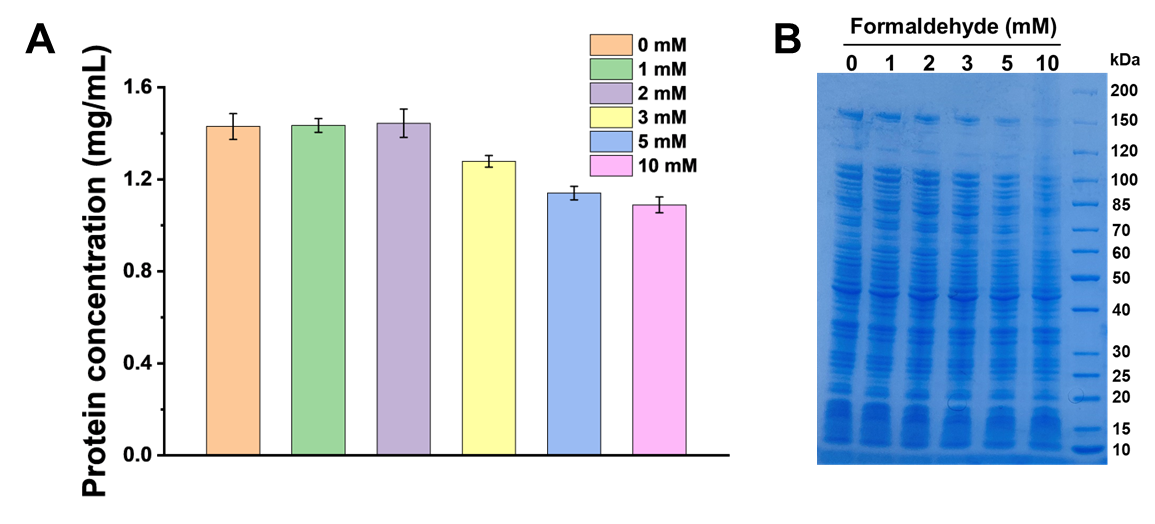


### Supplementary Figure **6. Formaldehyde-induced protein damage.** (A) Evaluation of protein concentration in the supernatant of strain MG1655 following a 10-hour exposure to 1-10 mM formaldehyde. (B) Analysis of proteins via SDS-PAGE in the supernatant of strain MG1655 post 10-hour treatment with 1-10 mM formaldehyde.


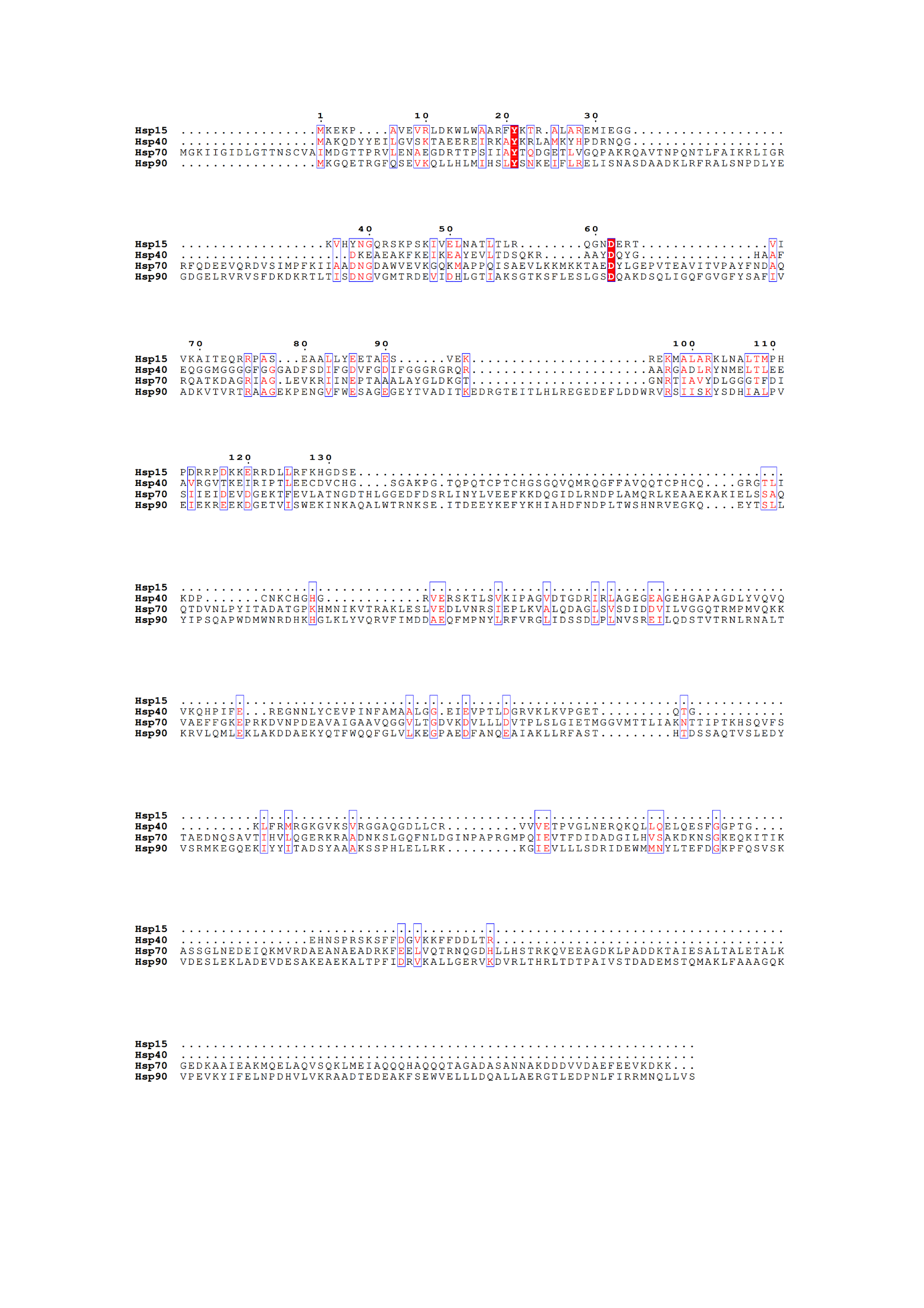


### Supplementary Figure 7. **Primary sequence alignment of proteins involved in alleviating protein aggregation.** Hsp15, Hsp40, Hsp70 and Hsp90 from *Escherichia coli*. The sequence identity is 8.5%.

**
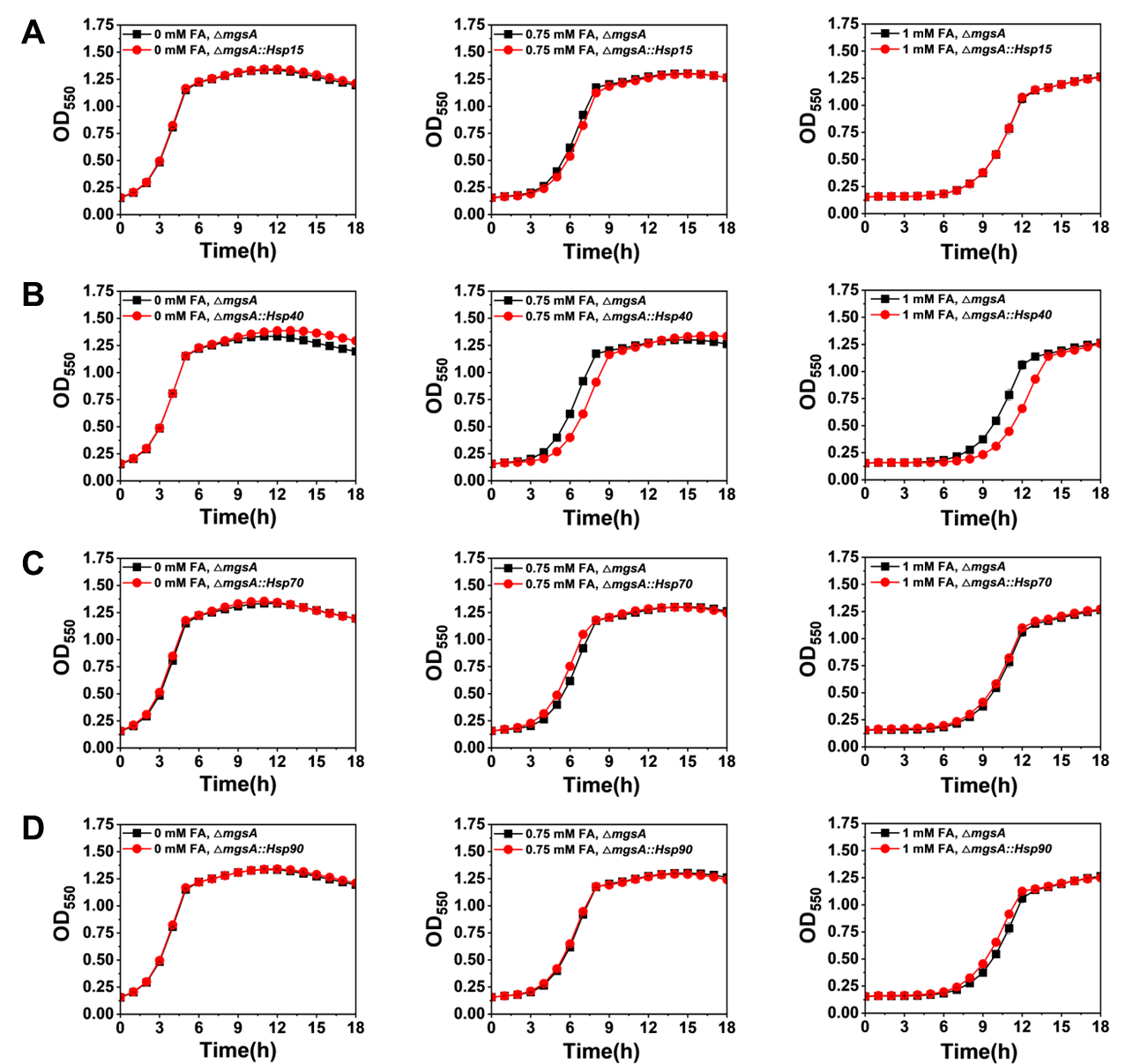
**

### Supplementary Figure 8. Formaldehyde tolerance of engineered strains utilized to detoxify protein aggregation. (A) – (D) *Hsp15, Hsp40, Hsp70* and *Hsp90* from *Escherichia coli* were inserted into the *mgsA* site. The strains were cultured in MOPS supplemented with 2% (wt/v) glucose containing 0 mM, 0.75 mM, and 1 mM formaldehyde in a clear-bottom 96-well plate at 37℃ and an initial pH of 7.0. Values are average of at least three biological replicates, and error bars indicate standard deviation.


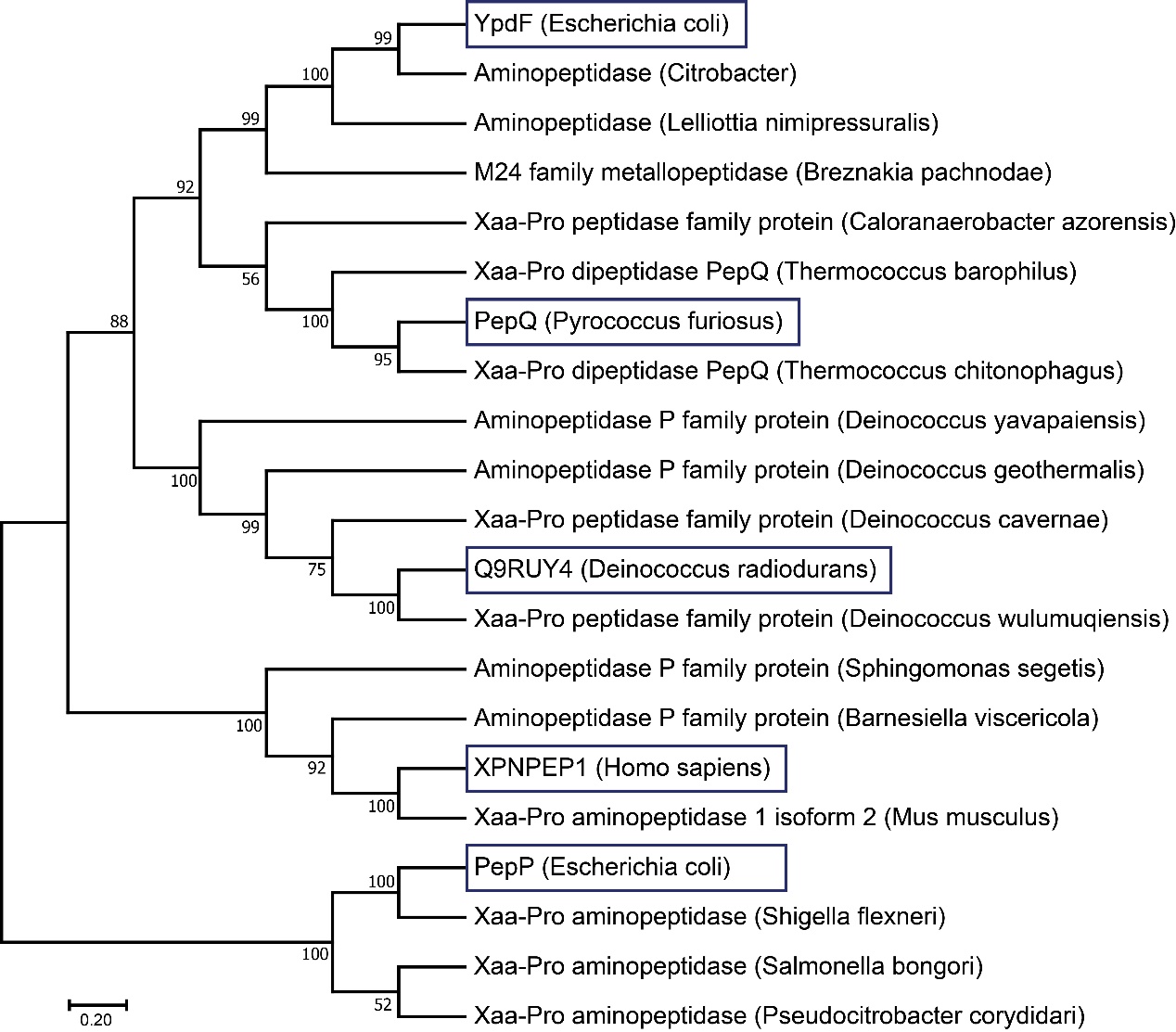


### Supplementary Figure 9. **Molecular phylogenetic analysis of proteins involved in alleviating thioproline damage.**


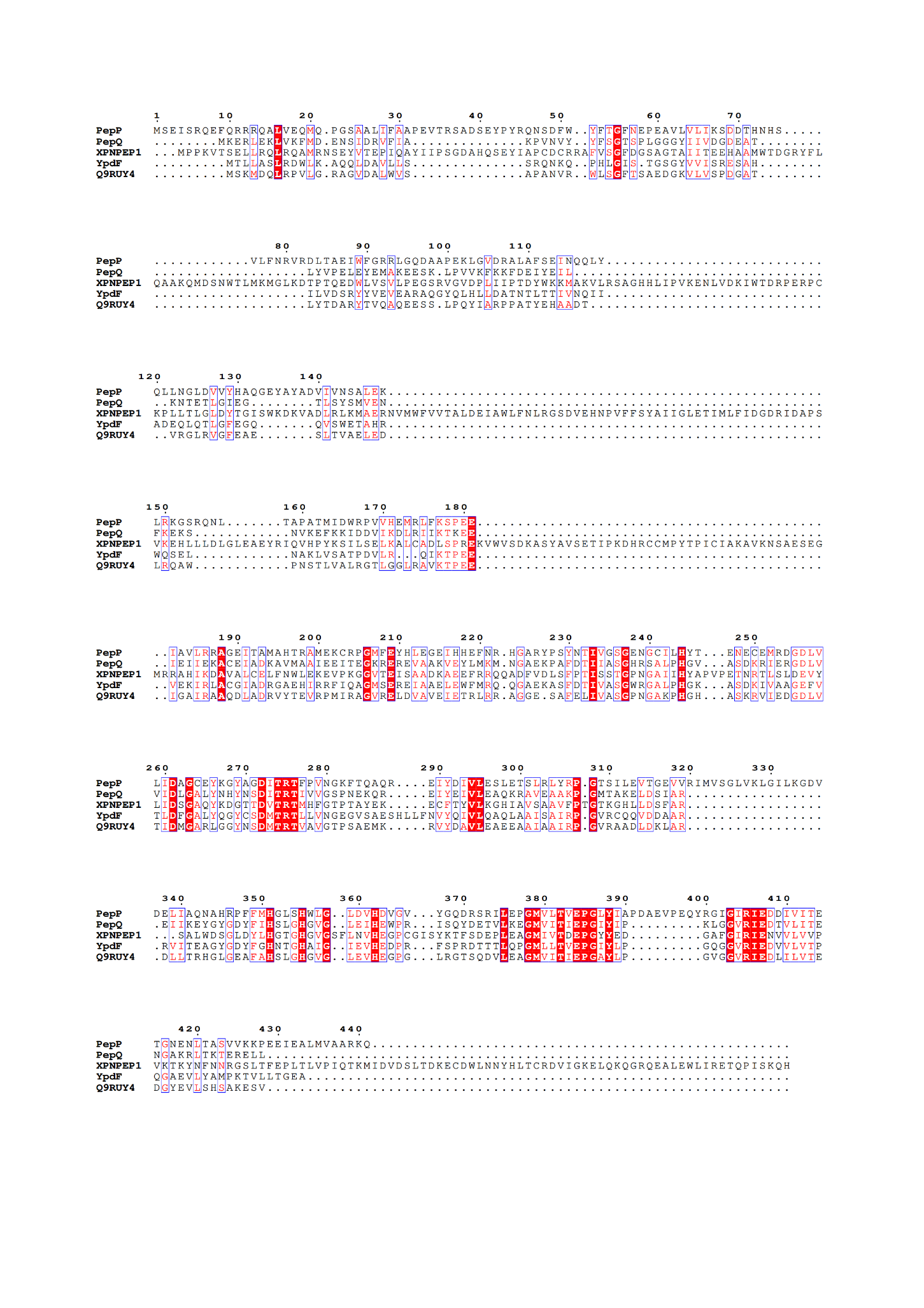


### Supplementary Figure 10. **Primary sequence alignment of proteins involved in alleviating thioproline damage.** PepQ from *Pyrococcus furiosus*, XPNPEP1 from *Homo sapiens*, Q9RUY4 from *Deinococcus radiodurans*, PepP and YpdF from *Escherichia coli*. The sequence identity is 22.4%.


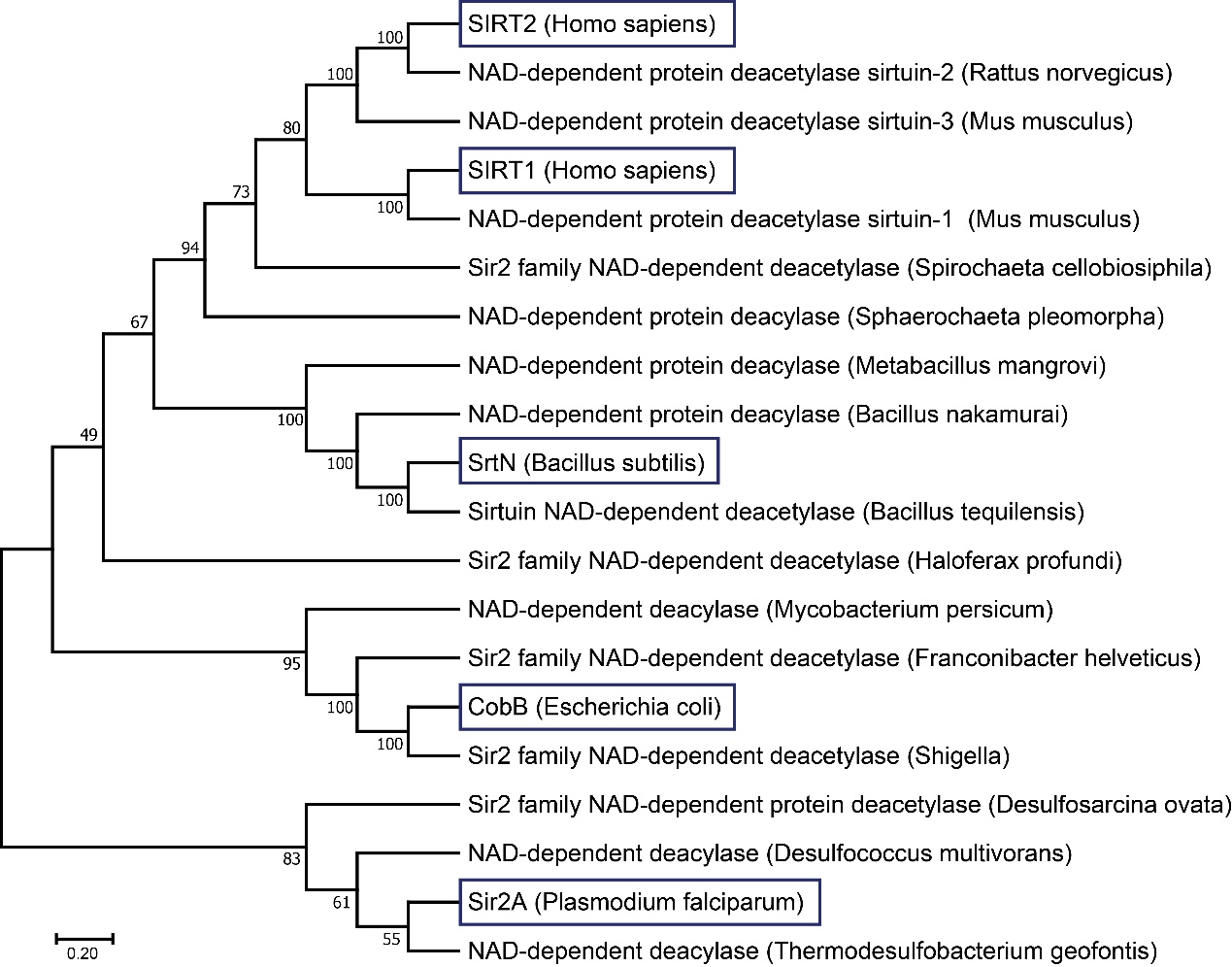


### Supplementary Figure 11. **Molecular phylogenetic analysis of proteins involved in alleviating *N*^6^-formyl-lysine damage.**


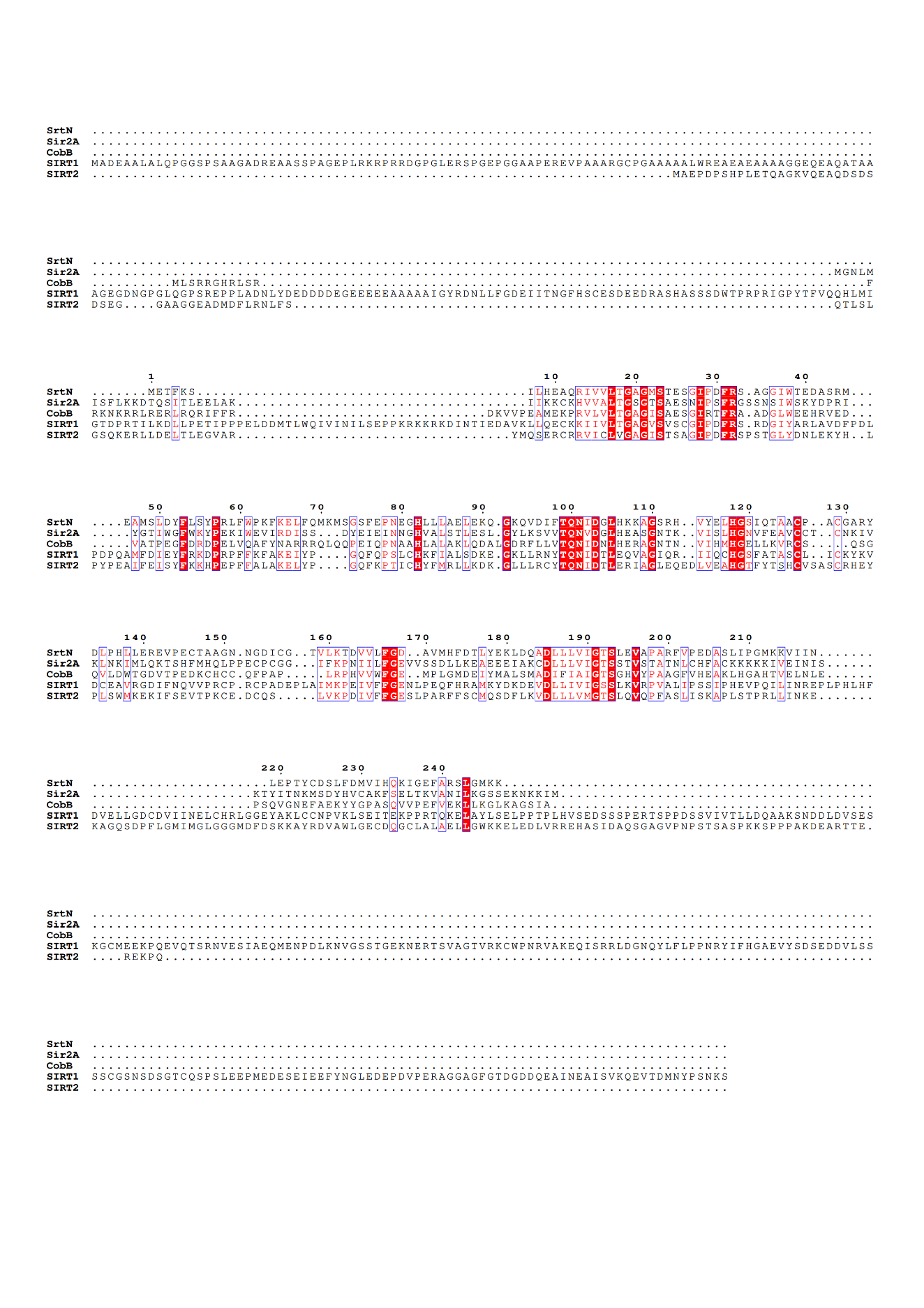


### Supplementary Figure 12. **Primary sequence alignment of proteins involved in alleviating N^6^-formyl-lysine damage.** SrtN from *Bacillus subtilis*, Sir2A from *Plasmodium falciparum*, CobB from *Escherichia coli*, SIRT1 and SIRT2 from *Homo sapiens*. The sequence identity is 19.6%.


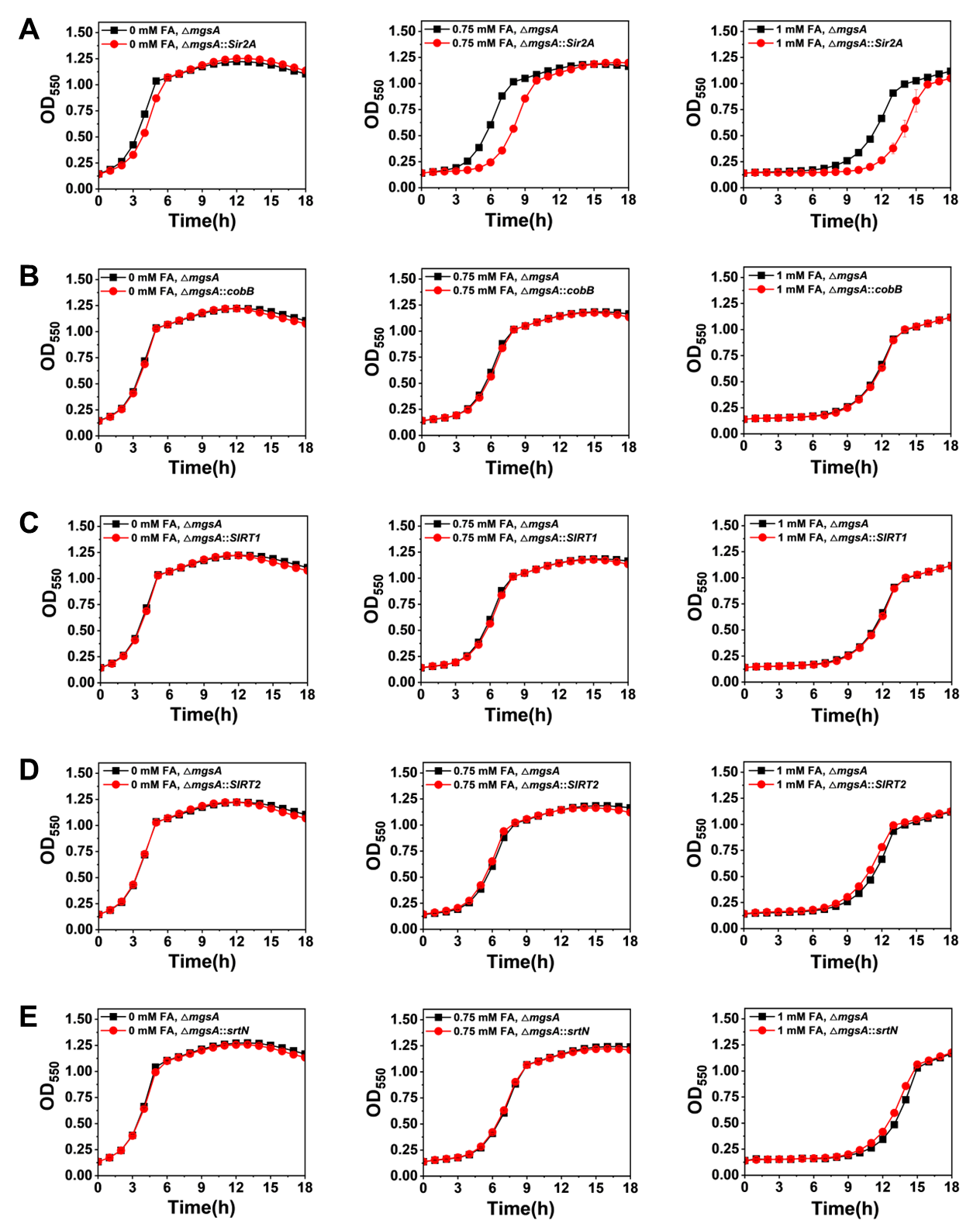


### Supplementary Figure 13. Formaldehyde tolerance of engineered strains utilized to detoxify *N*^6^-formyl-lysine. (A) – (E) Genes *Sir2A, cobB, SIRT1, SIRT2* and *srtN* were inserted into the *mgsA* site. The strains were cultured in MOPS supplemented with 2% (wt/v) glucose containing 0 mM, 0.75 mM, and 1 mM formaldehyde in a clear-bottom 96-well plate at 37℃ and an initial pH of 7.0. Values are average of at least three biological replicates, and error bars indicate standard deviation.


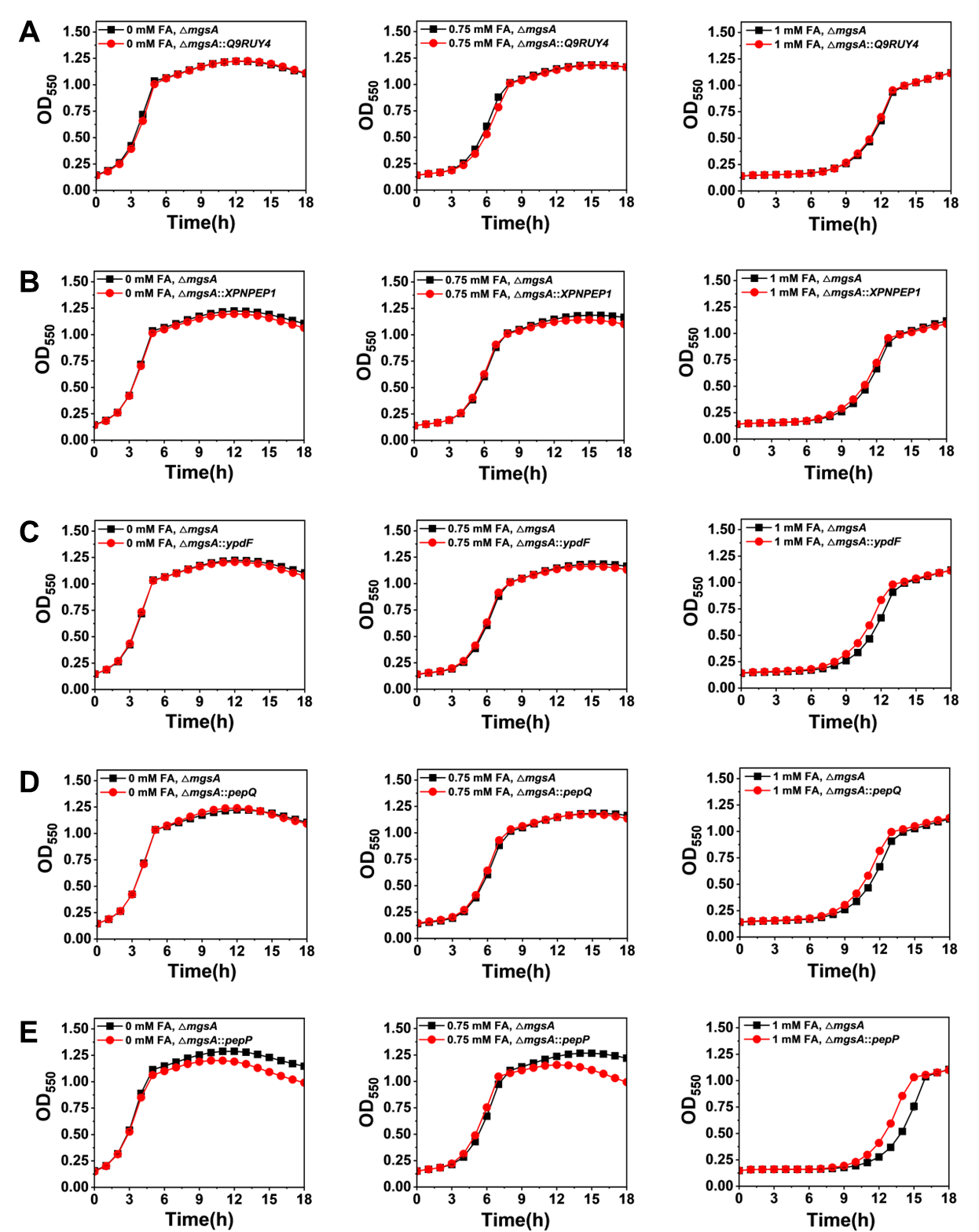


### Supplementary Figure 14. Formaldehyde tolerance of engineered strains utilized to detoxify thioproline. (A) – (E) Genes *Q9RUY4, XPNPEP1, ypdF, pepQ* and *pepP* were inserted into the *mgsA* site. The strains were cultured in MOPS supplemented with 2% (wt/v) glucose containing 0 mM, 0.75 mM, and 1 mM formaldehyde in a clear-bottom 96-well plate at 37℃ and an initial pH of 7.0. Values are average of at least three biological replicates, and error bars indicate standard deviation.


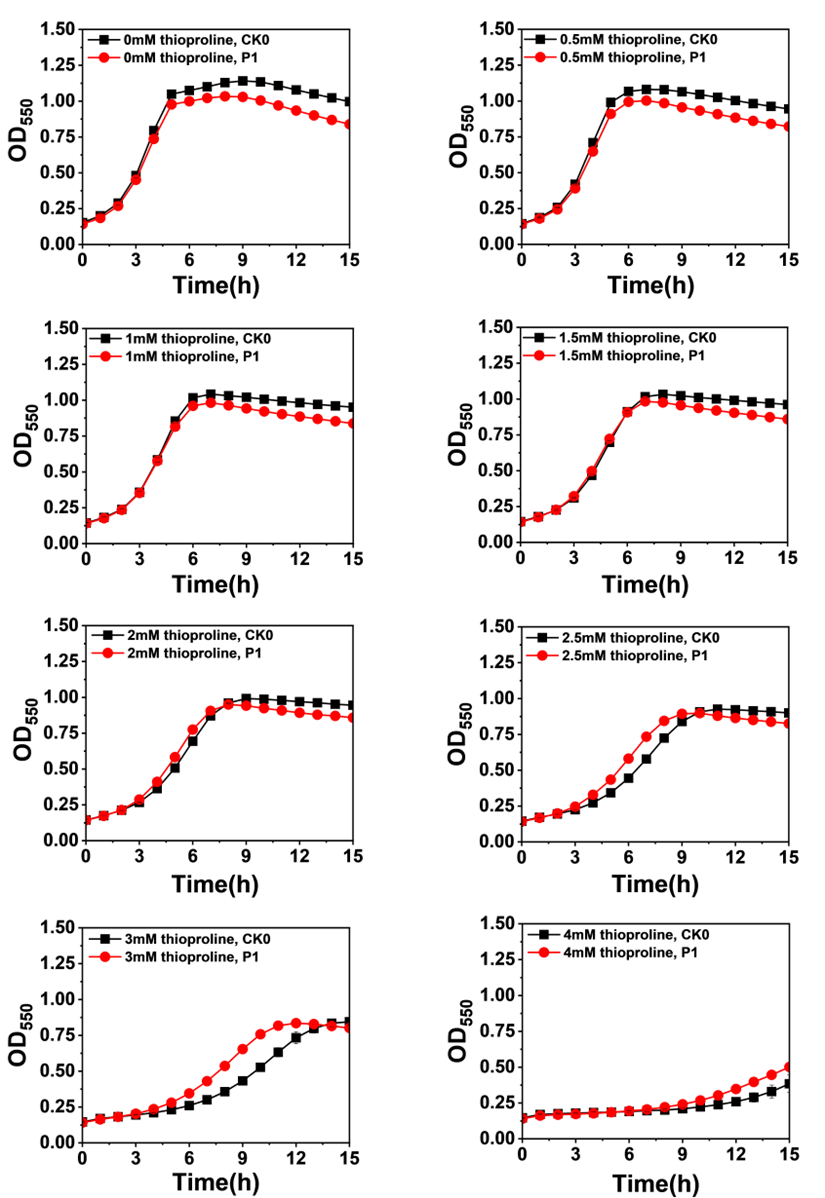


### Supplementary Figure 15. The growth of CK0 and P1 strains was evaluated in the presence of 0 to 4 mM thioproline in MOPS supplemented with 2% (wt/v) glucose, using a clear-bottom 96-well plate incubated at 37°C with an initial pH of 7.0. Error bars indicate standard error of the mean (n = 3).


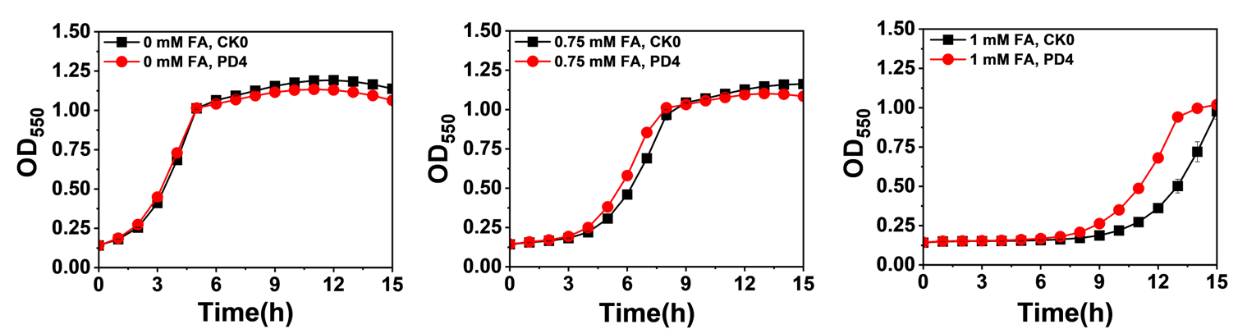


### Supplementary Figure 16. Formaldehyde tolerance of engineered strain PD4. Growth of strains in the presence of 0 mM, 0.75 mM, and 1 mM formaldehyde in MOPS+2% (wt/v) glucose in a clear-bottom 96-well plate at 37℃ and an initial pH of 7.0. Values are average of at least three biological replicates, and error bars indicate standard deviation.


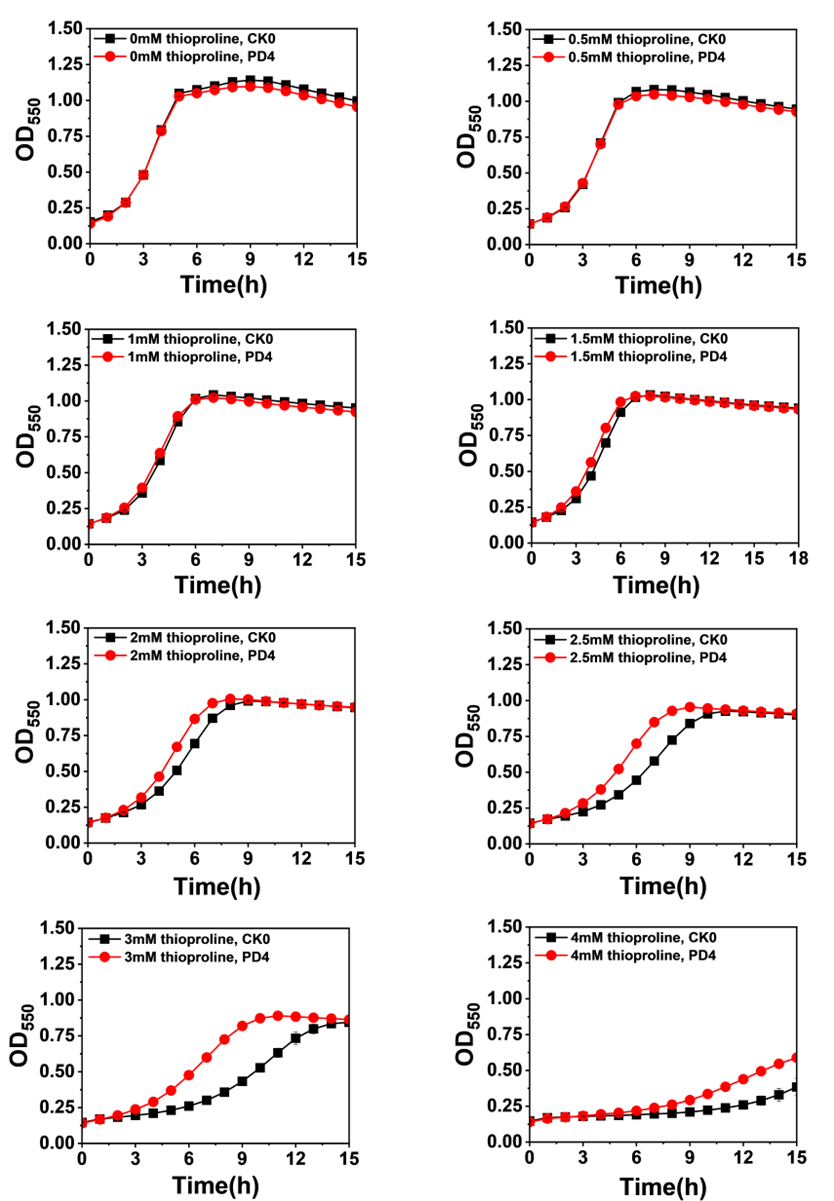


### Supplementary Figure 17. The growth of CK0 and PD4 strains was evaluated in the presence of 0 to 4 mM thioproline in MOPS supplemented with 2% (wt/v) glucose, using a clear-bottom 96-well plate incubated at 37°C with an initial pH of 7.0. Error bars indicate standard error of the mean (n = 3).


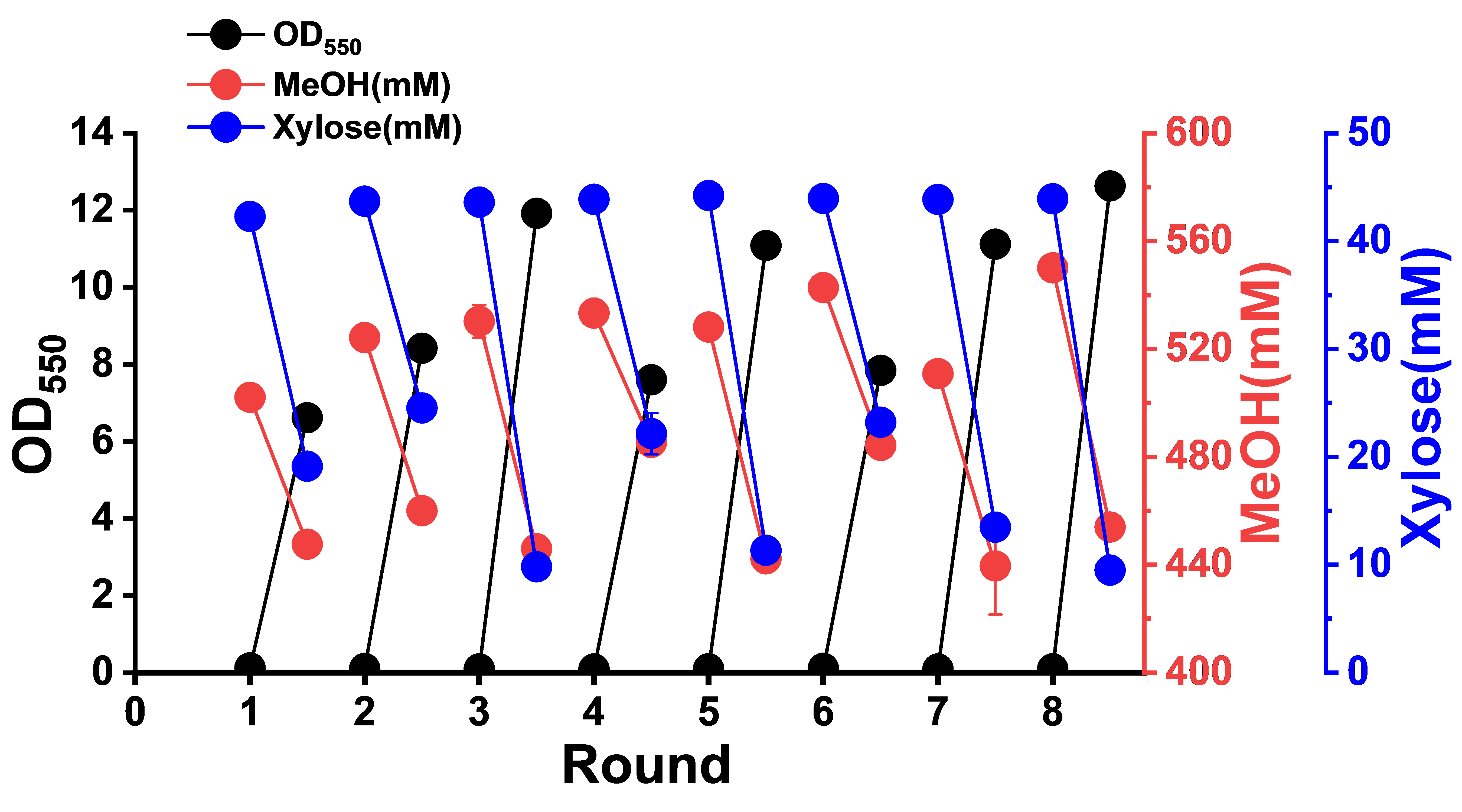


### Supplementary Figure 18. Growth dynamics of the MPD4 strain under the cultivation conditions of 40 mL MOPS medium containing 500 mM methanol and 50 mM xylose in 250 mL baffled flasks. Each passage was initiated at an OD_550_ of 0.1, and biomass accumulation alongside methanol and xylose consumption were quantified after 24 h of incubation, with the entire procedure repeated for eight consecutive passages. Error bars indicate standard error of the mean (n = 3).

[1] W.B. Whitaker, J.A. Jones, R.K. Bennett, et al. Engineering the biological conversion of methanol to specialty chemicals in *Escherichia coli*, *Metab. Eng.,* 39 (2017) 49-59. https://doi.org/10.1016/j.ymben.2016.10.015.

[2] W. Zhang, T. Zhang, M. Song, et al. Metabolic engineering of *Escherichia coli* for high yield production of succinic acid driven by methanol, *ACS Synth. Biol.,* 7 (2018) 2803-2811. https://doi.org/10.1021/acssynbio.8b00109.

[3] X. Wang, Y. Wang, J. Liu, et al. Biological conversion of methanol by evolved *Escherichia coli* carrying a linear methanol assimilation pathway, *BIOB,* 4 (2017) 1-6. https://doi.org/10.1186/s40643-017-0172-6.

[4] J.V. Price, L. Chen, W.B. Whitaker, et al. Scaffoldless engineered enzyme assembly for enhanced methanol utilization, *Proc. Natl. Acad. Sci. U.S.A.,* 113 (2016) 12691-12696. https://doi.org/10.1073/pnas.1601797113.

[5] C.T. Chen, F.Y.H. Chen, I.W. Bogorad, et al. Synthetic methanol auxotrophy of *Escherichia coli* for methanol-dependent growth and production, *Metab. Eng.,* 49 (2018) 257-266. https://doi.org/10.1016/j.ymben.2018.08.010.

[6] F Guo, M. Wu, S. Zhang, et al. Improved succinic acid production through the reconstruction of methanol dissimilation in *Escherichia coli*, *Bioresour. Bioprocess.,* 9 (2022) 1-13. https://doi.org/10.1186/s40643-022-00547-x.

[7] Q. Sun, D.H. Liu, C. Zhen, Engineering and adaptive laboratory evolution of *Escherichia coli* for improving methanol utilization based on a hybrid methanol assimilation pathway, *Front. Bioeng. Biotechnol.,* 10 (2023) 1089639. https://doi.org/10.3389/fbioe.2022.1089639.

[8] R.K. Bennett, J.E. Gonzalez, W.B. Whitaker, et al. Expression of heterologous non-oxidative pentose phosphate pathway from *Bacillus methanolicus* and phosphoglucose isomerase deletion improves methanol assimilation and metabolite production by a synthetic *Escherichia coli* methylotroph, *Metab. Eng.,* 45 (2018) 75-85. https://doi.org/10.1016/j.ymben.2017.11.016.

[9] M.K. Li, W.J. Sun, X. Wang, et al. A eukaryote-featured membrane phospholipid enhances bacterial formaldehyde tolerance and assimilation of one-carbon feedstocks, *Acs Synth Biol*., 13 (2024) 4074-4084. https://doi.org/10.1021/acssynbio.4c00499.
